# Multi-triangles cylindrical origami and inspired metamaterials with tunable stiffness and stretchable robotic arm

**DOI:** 10.1093/pnasnexus/pgad098

**Published:** 2023-03-23

**Authors:** Xiaolei Wang, Haibo Qu, Xiao Li, Yili Kuang, Haoqian Wang, Sheng Guo

**Affiliations:** Robotics Research Center, School of Mechanical, Electronic and Control Engineering, Beijing Jiaotong University, Beijing 100044, PR China; Research Center of Robotics Technology and Equipment, Tangshan Research Institute, Beijing Jiaotong University, Tangshan 063000, China; Robotics Research Center, School of Mechanical, Electronic and Control Engineering, Beijing Jiaotong University, Beijing 100044, PR China; Research Center of Robotics Technology and Equipment, Tangshan Research Institute, Beijing Jiaotong University, Tangshan 063000, China; Key Laboratory of Vehicle Advanced Manufacturing, Measuring and Control Technology, Ministry of Education, Beijing Jiaotong University, Beijing, China; Robotics Research Center, School of Mechanical, Electronic and Control Engineering, Beijing Jiaotong University, Beijing 100044, PR China; Research Center of Robotics Technology and Equipment, Tangshan Research Institute, Beijing Jiaotong University, Tangshan 063000, China; Robotics Research Center, School of Mechanical, Electronic and Control Engineering, Beijing Jiaotong University, Beijing 100044, PR China; Research Center of Robotics Technology and Equipment, Tangshan Research Institute, Beijing Jiaotong University, Tangshan 063000, China; Robotics Research Center, School of Mechanical, Electronic and Control Engineering, Beijing Jiaotong University, Beijing 100044, PR China; Research Center of Robotics Technology and Equipment, Tangshan Research Institute, Beijing Jiaotong University, Tangshan 063000, China; Robotics Research Center, School of Mechanical, Electronic and Control Engineering, Beijing Jiaotong University, Beijing 100044, PR China; Research Center of Robotics Technology and Equipment, Tangshan Research Institute, Beijing Jiaotong University, Tangshan 063000, China; Key Laboratory of Vehicle Advanced Manufacturing, Measuring and Control Technology, Ministry of Education, Beijing Jiaotong University, Beijing, China

**Keywords:** Kresling pattern origami, multistability, truss model, metamaterial, robotic arm

## Abstract

Kresling pattern origami-inspired structural design has been widely investigated using its bistable property and the single coupling degree of freedom (DOF). In order to obtain new properties or new origami-inspired structures, it needs to innovate the crease lines in the flat sheet of Kresling pattern origami. Here, we present a derivative of Kresling pattern origami—multi-triangles cylindrical origami (MTCO) with tristable property. The truss model is modified based on the switchable active crease lines during the folding motion of the MTCO. Using the energy landscape obtained from the modified truss model, the tristable property is validated and extended to Kresling pattern origami. Simultaneously, the high stiffness property of the third stable state and some special stable states are discussed. In addition, MTCO-inspired metamaterials with deployable property and tunable stiffness, and MTCO-inspired robotic arms with wide movement ranges and rich motion forms are created. These works promote research on Kresling pattern origami, and the design ideas of the metamaterials and robotic arms play a positive role in improving the stiffness of deployable structures and conceiving motion robots.

**Significance Statement**

Existing research claims that Kresling pattern origami has bistable property, and the analytical results of the general truss model validate this conclusion. Here, we create a new structure of Kresling pattern origami with tristable property by innovating the flat sheet of the triangulated cylindrical origami, which is named multi-triangles cylindrical origami. The tristable property is validated by the modified truss model, and the high stiffness of the third stable state is presented. Then, these properties are extended to the traditional Kresling pattern origami. Based on the aforementioned properties, origami-inspired metamaterials with tunable stiffness and stretchable origami robotic arm are studied. The tristable property and its high stiffness play an important role in research on Kresling pattern origami and origami-inspired structures.

Origami has received considerable attention from engineers and researchers, as a method for conceiving and analyzing new structures and materials (1). Origami-inspired structural design not only realizes complex shape transformation between space and plane but also obtains some mechanical properties that are difficult or impossible to achieve in traditional material design and synthesis (2, 3). Miura origami (4–6) with single degree of freedom (DOF) is one of the most widely used modes of rigid origami, which has been proven to have large volume variations, negative Poisson’s ratio, and anisotropic stiffness (6–9). Kresling pattern origami (10) with compression-torsion coupling DOF is one of the representative modes of nonrigid origami. It has multiple stable states, high stiffness, zero stiffness, high-density ratio, and great cushioning properties (1, 2, 11–14). Origami-inspired structures have been widely used in engineering fields, including robots (15–17), biomedical devices (18, 19), nanostructures (20, 21), aerospace (22, 23), and mechanical metamaterials (8, 24–27). Meanwhile, origami patterns are essentially metamaterials (28). Therefore, from the perspective of origami pattern expansion, it is of far-reaching significance to carry out in-depth research to promote the application of origami research in more engineering fields.

One of the most important concepts for origami-inspired structures is elastic energy landscape, which describes how strain energy varies with different geometries and/or shapes in the deformation configuration space (29). In rigid origami, elastic energy is stored at the creases and joints. In nonrigid origami, elastic energy is stored not only in the creases and joints but also in the panels. Thus, nonrigid origami offers more programmability in force, stiffness, and stability while providing complex deformation processes and energy landscapes. There are numerous studies have analyzed the folding motion by simulating the crease behavior (30–35), a relatively prominent one is the finite element method based on the truss folding structure (2, 13, 16, 25, 36–42), which can explicitly capture the crease behavior. However, the truss model in previous research cannot completely correspond to the folding motion, which is also why Kresling pattern origami is claimed to have bistable property rather than tristable property.

In this paper, the multi-triangles cylindrical origami (MTCO) with tristable property is presented by adding mountain crease lines into the triangulated cylindrical origami (TCO). Next, based on the switchable features between the mountain and valley crease lines, the folding motion is divided into two stages for modifying the truss model to obtain more accurate energy landscape. Through the local extreme points of the energy landscape, the tristable property of the MTCO is validated, and the high stiffness of the third stable state is pointed out. Then, the tristable property of the MTCO is extended to Kresling pattern origami, and some special stable states during the folding process are discussed. Finally, MTCO-inspired metamaterials are designed, possessing excellent deployable property and tunable stiffness. The load-bearing capacity of the metamaterials in multiple forms is validated experimentally. Compared to the robotic arm consisting of Kresling pattern origami, the MTCO-inspired robotic arm has richer stretchable ranges and movement forms. We envision that the proposed tristable property of Kresling pattern origami can provide new content for the design of deployable structures. MTCO-inspired metamaterials and robotic arms offer ideas for deployable structures to improve the load-bearing capacity and robots to achieve richer movement forms.

## Results

### Geometry of the multi-triangles cylindrical origami

**Fig. 1.**
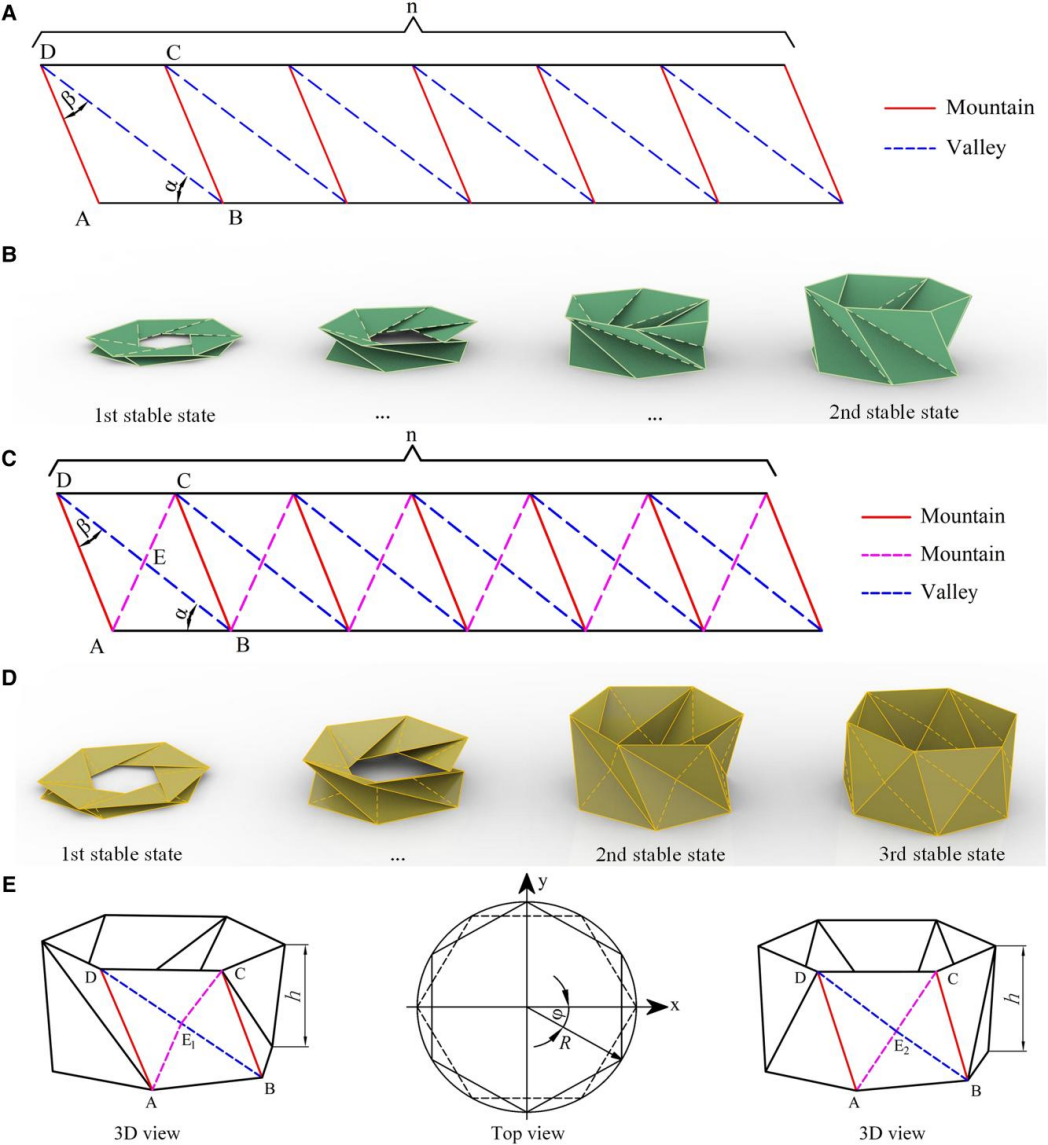
Geometry of the TCO and MTCO. A) The flat sheet with crease patterns consisting of mountain crease and valley crease lines of the TCO. B) Folding motion of the TCO, demonstrating two stable states. C) The flat sheet with crease patterns consisting of mountain crease and valley crease lines of the MTCO. D) Folding motion of the MTCO, demonstrating three stable states. E) The geometrical parameters of the MTCO during the folding motion.

The TCO in previous research is composed of repeated triangular arrays with one valley crease line and one mountain crease line in each cell. The flat sheet of the TCO is shown in Fig. 1A, the spatial folding motion is shown in Fig. 1B, and the focus is on its monostable, bistable, or zero-stiffness properties (1, 10, 25, 36). Here, MTCO is presented by adding a mountain crease line into each TCO cell, which is a derivative of Kresling pattern origami, as shown in Fig. S1. The flat sheet of the MTCO is shown in Fig. 1C, and the spatial folding motion is shown in Fig. 1D. The main features of the MTCO are that the design of the flat sheet expands the folding range compared to the TCO and realizes another stable state during the folding motion, which is defined as the third stable state. Movie S1 shows the structural characteristics and folding process of the MTCO.

Three parameters can be used to characterize the flat sheet of the MTCO, namely the bottom (top) polygon side length, *a* and two angles, *α* and *β*. There is *L*_*AB*_ = *a*, and the lengths of the crease lines at the planar state can be given by *L*_*AD*_ = *a*sin*α*/sin*β*, *L*_*BD*_ = *a*sin (*α* + *β*)/sin*β*, and $L_{AC}=a\sqrt{1+(\sin\;\alpha /\sin\;\beta {)}^{2}-2\sin\;\alpha \cos\;(\alpha +\beta )/\sin\;\beta }$. Similarly, the folding motion of the MTCO can be characterized by the height *h*, torsion angle *φ*, and radius *R*, as shown in Fig. 1E and Fig. S2. During the folding process, the valley crease line *BD* and mountain crease line *AC* in each cell switch with each other, but the mountain crease line *AD* remains maintained. The intersection of crease lines *BD* and *AC* is defined as *E*. In Fig. 1D and E, the first folding process is defined from the first stable state to the second one. During this process, point *E*_1_ is used to represent the midpoint of valley crease line *BD*, mountain crease line *AC* is divided into *AE*_1_ and *CE*_1_, and the valley crease line *BD* is the active crease line. The second folding process is defined from the second to third stable states. During this process, point *E*_2_ is used as a moving point between the midpoint of crease line *BD* and midpoint of crease line *AC*. The valley crease line *BD* is divided into *BE*_2_ and *DE*_2_, and the mountain crease line *AC* is divided into *AE*_2_ and *CE*_2_ (Table S1). Until the third stable state is formed, the mountain crease line *AC* becomes the active crease line, and the unit cell is divided into two triangular planes, as detailed in Fig. S3.

During the folding motion, the length of each crease line can be calculated by constructing a coordinate system, which are *l*_*AB*_ = 2*R*sin (*π*/*n*) and $l_{AD}=\sqrt{h^{2}-2R^{2}\cos\;\varphi +2R^{2}}$. During the first folding process, there are $l_{AC\_1}=l_{AE_{1}}+l_{CE_{1}}$ and $l_{BD\_1}=\sqrt{h^{2}-2R^{2}\cos\;(2\pi /n+\varphi )+2R^{2}}$. During the second folding process, there are $l_{AC\_2}=l_{AE_{2}}+l_{CE_{2}}$ and $l_{BD\_2}=l_{BE_{2}}+l_{DE_{2}}$ (_1 and _2 represent the first and second folding processes, respectively). Detailed geometric calculations are available in *SI Note* 1.

### Truss model of the multi-triangles cylindrical origami

The truss model is widely used as a method to qualitatively analyze the multistable property of Kresling pattern origami (1, 10, 25, 36, 41–44). The main idea of the truss model is to ignore the panel deformation during the folding motion, assuming that the changes in elastic energy are caused by stretching or shortening the crease lines. However, it should be noted that during the folding motion of Kresling pattern origami from the second stable state position, the crease lines gradually present an arc shape. At this time, there is an error in continuing to calculate the lengths of the crease lines with spatial coordinate points. This also explains why the energy rises sharply after the second stable state position. Here, the truss model is modified through the switchable feature of the active crease lines during the folding motion of the MTCO. The modified truss model captures the motion behavior of the crease lines more accurately and expresses another stable state.

The folding motion of the MTCO is divided into two processes based on the switchable feature between the mountain and valley crease lines. During the first folding process, the MTCO cell is divided into two triangular panels Δ*ABD* and Δ*BCD* by valley crease line *BD*, and mountain crease line *AC* can be represented by *AE*_1_ and *CE*_1_. During the second folding process, the MTCO cell is divided into four triangular panels Δ*ABE*_2_, Δ*BCE*_2_, Δ*CDE*_2_, and Δ*ADE*_2_ by valley crease line *BD* and mountain crease line *AC*. The valley crease line *BD* can be represented by *BE*_2_ and *DE*_2_, and the mountain crease line *AC* can be represented by *AE*_2_ and *CE*_2_ (Table S1). Similarly, the bottom and top polygons are still regarded as rigid. And using Δ*L*_*AD*_ = *l*_*AD*_ − *L*_*AD*_, Δ*L*_*BD*_ = *l*_*BD*_ − *L*_*BD*_, and Δ*L*_*AC*_ = *l*_*AC*_ − *L*_*AC*_ to represent the deformation of the three crease lines. In these two folding processes, the calculation methods of the crease lines *BD* and *AC* are different, which has been mentioned in the previous chapter. The MTCO cannot be stretched further when at the third stable state. Therefore, the boundary condition for the truss model is defined as $L_{AC}=l_{AC\_2}$. Details of the modified truss model can be observed in *SI Note* 2.

**Fig. 2.**
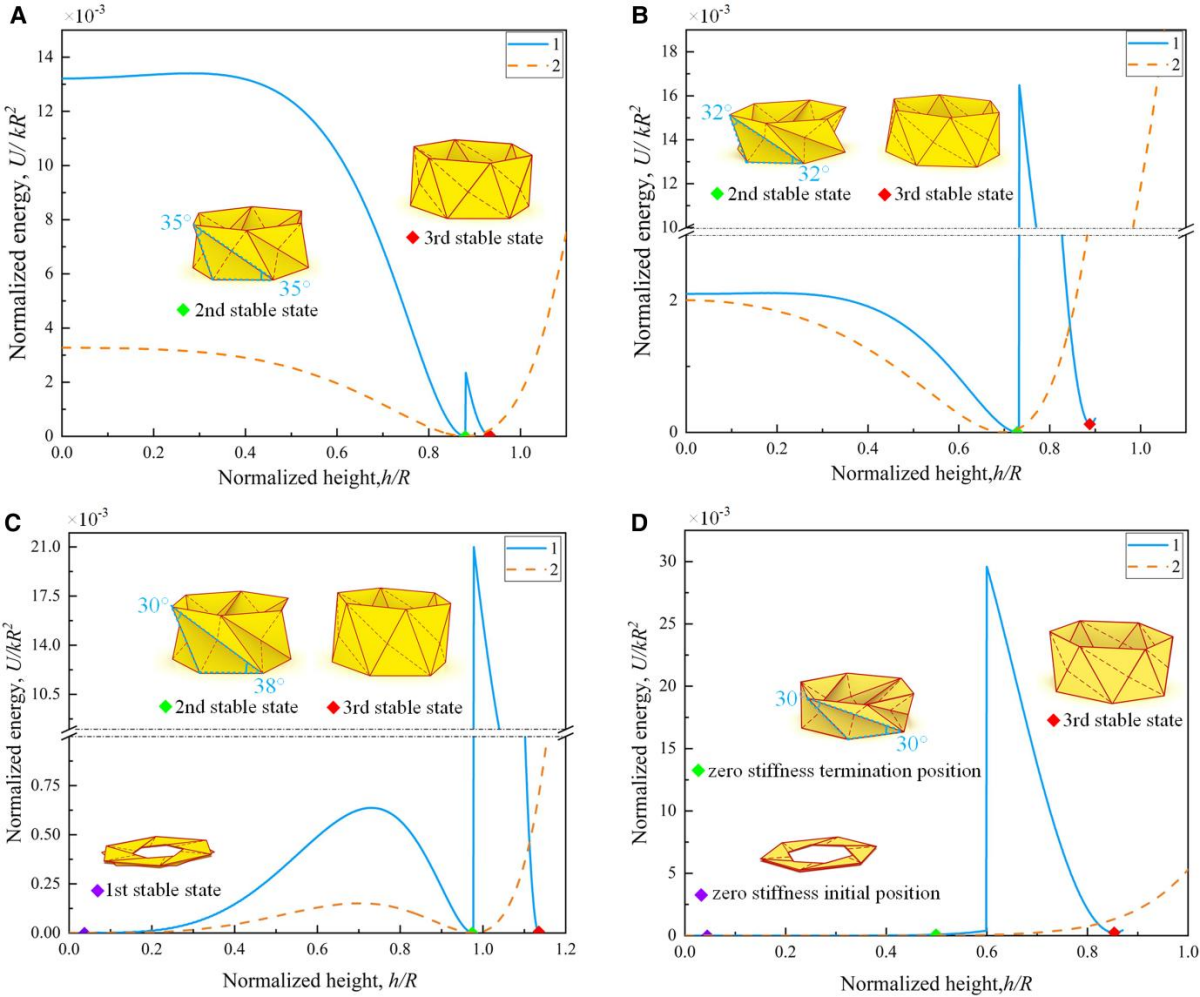
Energy landscapes of the MTCO during the folding motion. A–D) The energy analysis for the MTCO-based truss model shows remarkably different behaviors: A) Bistability at $\alpha =\beta =35^{\circ }$. B) Bistability at $\alpha =\beta =32^{\circ }$. C) Tristability at $\alpha =38^{\circ }$ and $\beta =30^{\circ }$. D) Zero-stiffness mode at $\alpha =\beta =30^{\circ }$. The insets show representative features of the MTCO during the folding motion, corresponding to the different colored markers in the energy landscapes. (1 denotes the results obtained from the modified truss model, and 2 denotes the results obtained from the previous truss model.)

The energy $U=1/2k(\Delta L_{AD}^{2}+\Delta L_{BD}^{2}+\Delta L_{AE_{1}}^{2}+\Delta L_{CE_{1}}^{2})$ stored in the MTCO cell during the first folding process can be obtained using the elastic potential energy theorem *U* = 1/2*k*Δ*L*^2^ (where *k* is the elastic constant of the linear truss element). The energy $U=1/2k(\Delta L_{AD}^{2}+\Delta L_{BE_{2}}^{2}+\Delta L_{DE_{2}}^{2}+\Delta L_{AE_{2}}^{2}+\Delta L_{CE_{2}}^{2})$ during the second folding process can also be calculated (Table S2). Then, the energy can be simplified as the normalized form (*U*/*kR*^2^), which is a function of *h* and *φ*. At this point, the correction for the truss model has been completed, as detailed in Figs. S4 and S5. Based on the modified truss model, several typical properties of the MTCO are analyzed by applying ∂*U*/∂*φ* = 0, as shown in Fig. 2. If $\alpha =\beta =35^{\circ }$, the energy landscape has only two minimum energy positions (Fig. 2A), meaning that the structure has bistable property. If $\alpha =\beta =32^{\circ }$, the energy landscape also has two minimum energy positions as shown in Fig. 2B, and the origami has bistable property. The MTCO with $\alpha =\beta =35^{\circ }$ is locally deployable, however it will be fully deployable when $\alpha =\beta =32^{\circ }$. According to the characteristics of the deployable property, the MTCO is classified into four cases (see *SI Note* 3), including bistable locally deployable, bistable fully deployable, tristable fully deployable, and zero stiffness fully deployable. And the applicability of the truss model is illustrated, as shown in Figs. S6 and S7. If $\alpha =38^{\circ }$ and $\beta =30^{\circ }$, the energy landscape has three local extreme points (Fig. 2C), indicating tristable property, which is an innovative discovery. If $\alpha =\beta =30^{\circ }$, the energy increases at an extremely low rate around *U* = 0 for a period of time (Fig. 2D), the MTCO has zero stiffness and the third stable state properties at the same time.

### Multiple properties of the multi-triangles cylindrical origami

**Fig. 3.**
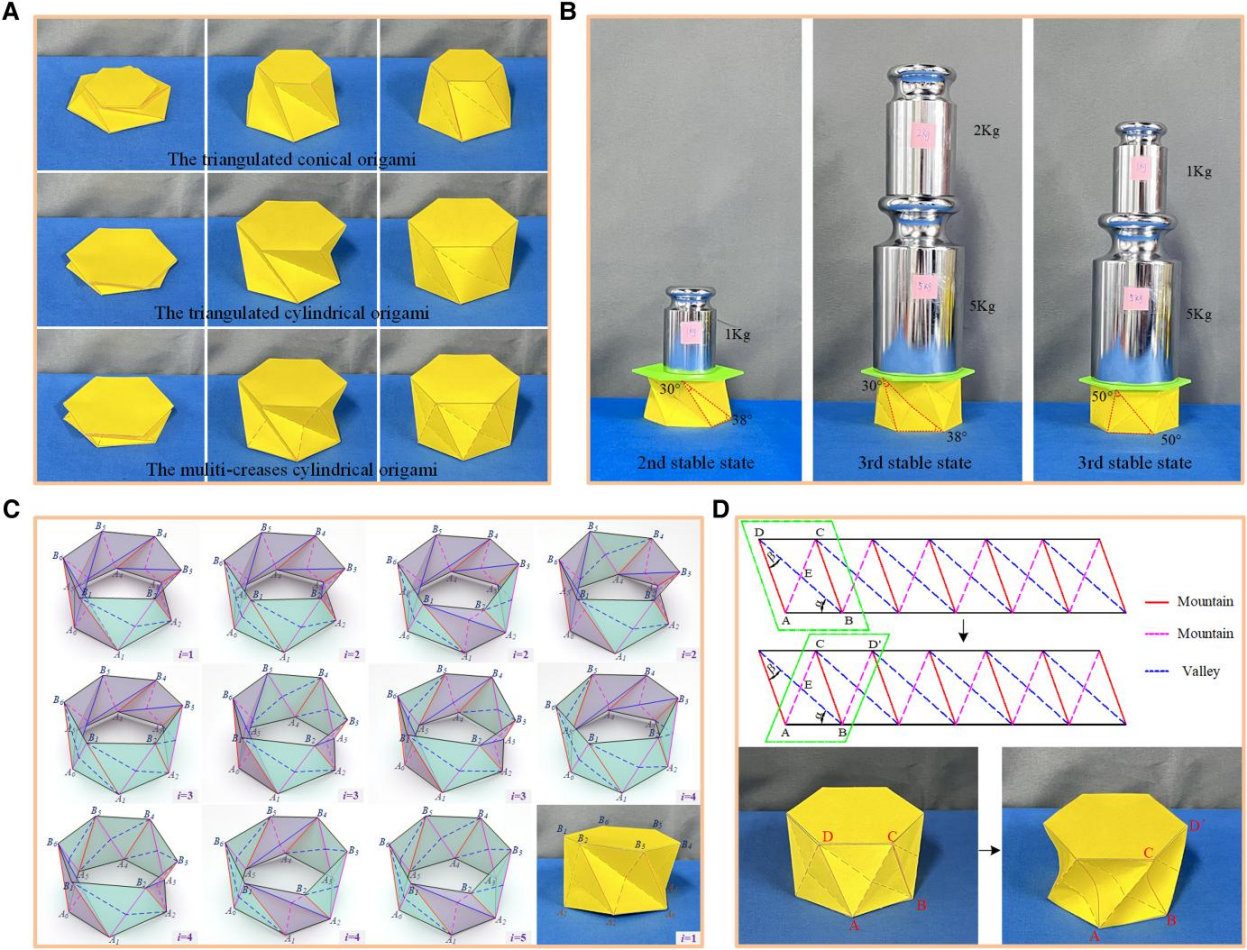
Multiple properties of the multi-triangles cylindrical origami. A) The three stable states of Kresling pattern origami, including the triangulated conical origami, triangulated cylindrical origami and multi-triangles cylindrical origami. B) The experimental validated photos of the high stiffness property of the third stable state. C) The special stable states that the top and bottom polygons exhibit large deformation (*i* denotes the number of unit cells with the form of the third stable state). D) Another stable state that the definition of the crease lines has changed and large deformation is exhibited in the unit cells.

The modified truss model reveals the tristable property of the MTCO, and the third stable state is also presented in the triangulated cylindrical origami and triangulated conical origami. In other words, Kresling pattern origami has tristable property, as shown in Fig. 3A (Movie S2). The second and third stable states always existed, which is consistent with the actual folding motion. The feature of the third stable state of the MTCO is that the unit cell is divided into two triangular surfaces Δ*ABC* and Δ*ACD* by mountain crease line *AC*. For the triangulated cylindrical origami and triangulated conical origami, the feature of the third stable state is that the unit cell presents a surface that curves outward as a whole, and the bending reaches a maximum along the diagonal line *AC* (see *SI Note* 4).

There are large energy obstacles shown in the energy landscapes (Fig. 2), indicating that the MTCO has high stiffness at the third stable state. The load-bearing capacity was taken as a measurement index to validate the high stiffness property, as shown in Fig. 3B. The weight that the origami sample can carry at the second stable state was 1 kg, and the consequences was 7 kg at the third stable state, demonstrating the high stiffness of the third stable state. Meanwhile, the accuracy of the modified truss model is also validated. Compared to $\alpha =\beta =50^{\circ }$, the MTCO with $\alpha =38^{\circ }$ and $\beta =30^{\circ }$ has higher stiffness at the third stable state. Owing to the inevitable error of manual origami samples, the unit of loads applied during the experiments was 1 kg, which can be observed in Movie S3. In addition, an idea to divide the MTCO into different forms with the geometric parameter $\alpha +\beta =90^{\circ }$ as the boundary is proposed (see *SI Note* 5 and Fig. S8). With this idea, the phenomenon that the definition of the crease lines of the MTCO at the second and third stable states is different from the one of the flat sheet can be explained.

During the folding process between the second stable state and the third one, the MTCO inevitably takes on some special stable states that both the forms of the second and third stable states exist between the unit cells. Whether the MTCO is at the second stable state or the third one, the top and bottom polygons always keep horizontal. However, for these special stable states, the top and bottom polygons exhibit irregular surfaces with large internal deformation. And if the deformation of the top and bottom polygons is constrained, it will be generated on the crease lines and planes, which may cause serious panel damage. Based on the number of unit cells with the form of the third stable state, there are 11 kinds of these special stable states (*n* = 6), as shown in Fig. 3C (Movie S4). A point-searching method is utilized to explain these special stable states (Fig. S9 and Tables S3–S5), which validates the modified truss model once again, as detailed in *SI Note* 6.

There is another interesting phenomenon during the folding process of the MTCO. When the MTCO is at the third stable state, if external forces are applied to the mountain crease lines *AD*, they will disappear into the unit cells, and the valley crease lines *BD* will be divided by mountain crease lines *AC*. Then the mountain crease lines *AC* become the main crease, and the MTCO will obtain another stable state. Although the mountain crease lines *AC* and valley crease lines *BD* switch with each other to form the third stable state, the definition of the crease lines does not change, and they always remain in the original cells. However, for the special stable state mentioned here, the definition of the mountain crease lines *AD* and valley crease lines *BD* has changed, and new unit cells have formed, as shown in Fig. 3D (Movie S5). The idea of the truss model is that the top and bottom polygons are assumed as rigid, and the panel deformation can be simplified in the crease lines. However, for these special stable states, there is a large amount of deformation on the top and bottom polygons or unit cells, therefore, it is difficult to analyze with the truss model.

### MTCO-inspired metamaterials with tunable stiffness

**Fig. 4.**
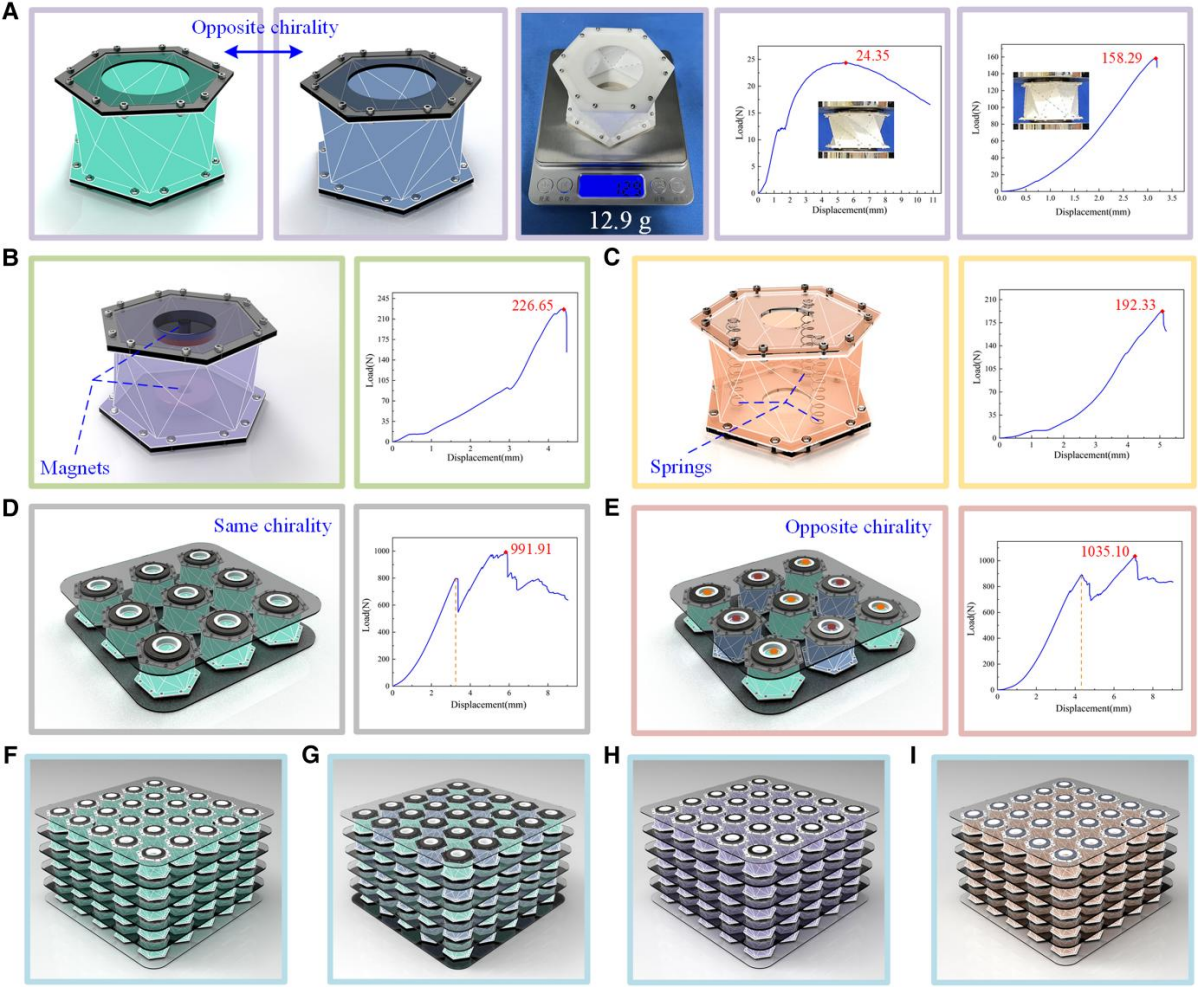
The metamaterials inspired by the multi-triangles cylindrical origami. A) Model of the single-module metamaterial with opposite chirality and the load/displacement curves. B) Model of the single-module metamaterial under opposite magnetic field and the load/displacement curve. C) Model of the single-module metamaterial with built-in springs and the load/displacement curve. D) Modular metamaterial with same chirality and the load/displacement curve. E) Modular metamaterial with opposite chirality and the load/displacement curve. F) Metamaterial array with same chirality (5 × 5 × 5). G) Metamaterial array with opposite chirality. H) Metamaterial array under opposite magnetic field. I) Metamaterial array with built-in springs.

Kresling pattern origami is not rigidly deployable, folding motions cause the warping and deformation of each facet, which may result in surface fatigue and damage under repeated usage (25). To overcome this problem while retaining the critical properties of the MTCO, polypropylene sheets with 0.5 mm thickness are used to create MTCO-inspired metamaterials ($\alpha =38^{\circ }$ and $\beta =30^{\circ }$). The metamaterials with deployable property and tunable stiffness are shown in Fig. 4 (Figs. S10 and S11 and Movie S6). The performance parameters of the polypropylene material are shown in Table 1 (see *SI Note* 8 and Fig. S12). In the research by Zhai (36), the high stiffness of the origami and metamaterial is attributed to the geometrical parameters ($\alpha +\beta \geq 90^{\circ }$), and Kresling pattern origami corresponding to these parameters is not deployable. However, in this study, the tunable stiffness of the metamaterials corresponds to the high stiffness property of the third stable state of Kresling pattern origami. Therefore, multiple design approaches are necessary to maintain the third stable state for improving the stiffness of the metamaterials.

**Table 1.** Mechanical properties of the polypropylene material.

Properties	Value
Young’s modulus (MPa)	869.6
Poisson’s ratio	0.345
Tensile strength (MPa)	22.36
Elongation	8%

Similar to Kresling pattern origami, the metamaterial also has different chirality, as shown in Fig. 4A. A universal tensile testing machine was used to test the load-bearing capacity of the metamaterials. The load/displacement curves obtained show that the peak load of the single-module metamaterial ($\alpha =38^{\circ },\beta =30^{\circ }$) at the second stable state was 24.35 N, and the maximum load was 158.29 N at the third stable state (Fig. S13), meaning that the load-bearing capacity was increased by 5.50 times. The weight of the single-module metamaterial was only 12.9 g, which means that it could carry a load of 1252 times its own weight. Two ring-shaped magnets were arranged opposite each other on the top/bottom polygon plates to increase the stiffness by magnetic repulsion, and the peak load was 226.65 N, as shown in Fig. 4B. If springs were placed inside the metamaterial to increase the stiffness as well, it had a maximum load of 192.33 N, as shown in Fig. 4C. It is conceivable that the stronger the magnetic field and the stiffer the spring, the more significant the improvement in the stiffness of the metamaterial. In addition, the modular metamaterials provide greater stiffness while accommodating different sizes, which can be arranged with the same or opposite chirality, as shown in Fig. 4D and E. The modular metamaterial with same chirality arrangement is shown in Fig. 4D, which had a maximum load of 991.91 N. And the modular metamaterial with opposite chirality is shown in Fig. 4E, in which the peak load was 1035.10 N (see *SI Note* 9). The single-module metamaterial was folded first during the compression process, causing stress concentration. Then the stress concentration would produce a torque effect on the surrounding modules and intensify the folding process of the modular metamaterial with the same chirality. However, the rotational directions between the modules of the modular metamaterial with opposite chirality are different, which would constrain the torque influence produced by the stress concentration (Fig. S14). It can be concluded that the opposite chirality arrangement can improve the stability of the modular metamaterials. Different metamaterial arrays (5 × 5 × 5) are shown in Fig. 4F–I, including arrangements of the same chirality, opposite chirality, built-in reverse magnetic fields, and built-in springs. Also, the combination of multiple arrangements can be extended to improve the stiffness and stability of the metamaterials.

### MTCO-inspired stretchable robotic arm

Kresling pattern origami with bistable property combined with magnetically responsive materials provides ideas for mobile robots and bionic robotic arms (11, 45). Modular origami can achieve stretching, folding, omnidirectional bending, and twisting motions when external forces are applied. However, MTCO with the third stable state provides a larger motion range and richer motion characteristics while improving stiffness, which offers new content for origami-inspired robots and robotic arms. For multistable metamaterials, multiple stable states during the folding motion are research interest. For robotic arms with multimodal motions, fewer deformations between multiple motion forms (small energy barriers during the folding process) are considered, which is beneficial for reducing the material damage with repeated motions. Therefore, Kresling pattern origami with zero stiffness property is more appropriate for robotic arms.

**Fig. 5.**
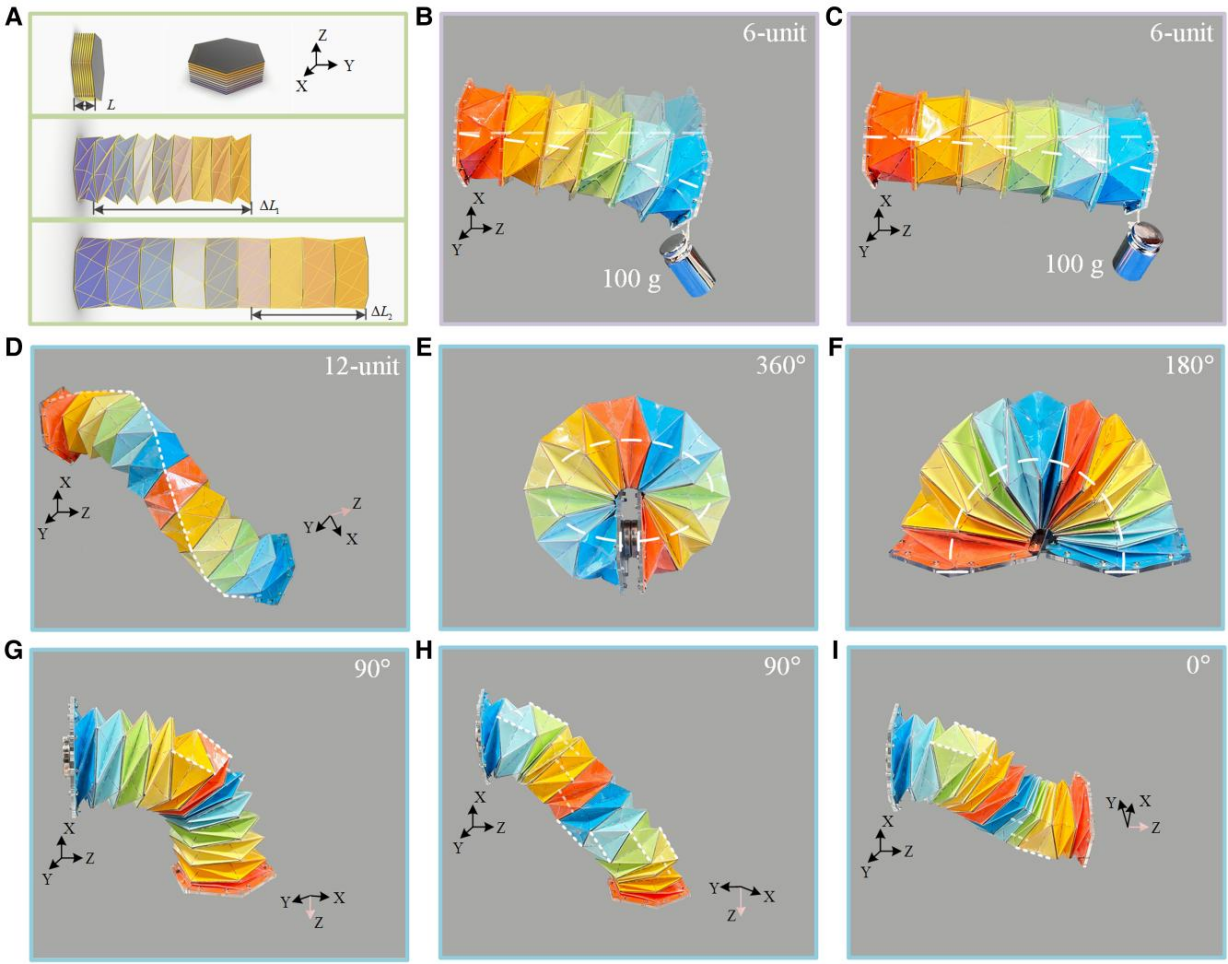
The robotic arm inspired by the multi-triangles cylindrical origami. A) The MTCO with the third stable state extends the stretchable range of the robotic arm (9-unit). B,C) Photos of stiffness validated on the robotic arm (6-unit), the suspended load was 100 g. B) The bending degree of the robotic arm at the second stable state (zero stiffness termination position). C) The bending degree of the robotic arm at the third stable state. D–I) Multiple motion forms of the robotic arms (12-unit). D) Free extension state of the robotic arm. The unit cells along the white dashed line are with the form of the third stable state, and the rest of the unit cells are with the form of the zero stiffness termination. E) The robotic arm achieves a bending angle of 360${}^{\circ }$. F) The robotic arm achieves a bending angle of 180${}^{\circ }$ (Fig. S20). G) The robotic arm achieves a bending angle of 90${}^{\circ }$, including three unit cells with the form of the third stable state. H) The robotic arm achieves a bending angle of 90${}^{\circ }$, including ten unit cells with the form of the third stable state distributed in a spiral pattern. I) The robotic arm achieves extending with a twisted manner.

Compared to the second stable state, the single-module MTCO ($\alpha =\beta =30^{\circ }$) with the third stable state expands the stretchable range by 66.9% and the bendable degree by 74.3% (see *SI Note* 10). When an external force is applied to bend the top and bottom of the MTCO, the maximum bending angle could reach 25.54${}^{\circ }$, as shown in Fig. S15. The stretchable range of the MTCO-inspired robotic arm is shown in Fig. 5A, and the outer surface of the cardboard was covered with a film to improve fatigue resistance (Figs. S16 and S17). The stiffness of the robotic arm was validated, as shown in Fig. 5B and C. For two stable states of the robotic arm (6-unit), there is a difference in the bending degree when hanging a 100 g weight, indicating that the MTCO-inspired robotic arm has better stiffness. Multiple motion modes of the robotic arm (12-unit) were simulated, as shown in Fig. 5D–I and Movie S7. The modular design and the top/bottom polygon reduction (Fig. S18) facilitate the deformation of the robotic arm, allowing a larger bending angle of approximately 37.17${}^{\circ }$ (Fig. S19). The robotic arm achieves a bending angle of 360${}^{\circ }$, as shown in Fig. 5E. In conclusion, the MTCO with the third stable state exhibits better stiffness and motion characteristics, which provides references for robotic arms and robots.

## Concluding remarks

In this work, we present a new structure of Kresling pattern origami—multi-triangles cylindrical origami from the perspective of origami pattern innovation and complete the modification of the truss model that can be used to analyze the motion behavior of the crease lines. On this basis, the tristable property of Kresling pattern origami is presented, and the high stiffness property of the third stable state is validated. The proposed tristable property and the modified truss model are innovative development in research on Kresling pattern origami. Simultaneously, some special stable states are mentioned, one of which is the change in the definition of the crease lines. Furthermore, MTCO-inspired metamaterials with deployable property, multi-stability, and tunable stiffness are designed, carrying a load of approximately 1252 times its own weight. The modular design concept yields better stiffness while allowing for different dimensions. In addition, the MTCO-inspired robotic arm demonstrates larger motion ranges and richer motion modes, which can inspire the design of robots and robotic arms. In conclusion, we believe that the research on the tristable property of Kresling pattern origami and the modification of the truss model in this work provide new content for the design of deployable structure, multi-stable metamaterial, and robotic arm.

## Materials and methods

### Prototype fabrication

In this study, the origami-inspired robotic arm samples were fabricated with 120 g/m^2^ cardboard and covered with a film on the outer surface. The rest of the origami samples were fabricated using 200 g/m^2^ cardboard. The metamaterials were made of polypropylene sheets with 0.5 mm thickness, and the top/bottom polygon panels were 3D printed resin. Then M1.4 * 5 mm bolts were used to connect the polypropylene pattern to the polygonal plates. More details are provided in *SI Note* 7.

### Performance testing

The load-bearing capability of the metamaterials were characterized using a universal tensile testing machine. The samples were placed between two fixtures of the testing machine. The load cells were 500 N/5,000 N, and the displacement rate was 2 mm/min. Multiple runs were conducted to achieve average measurement. For the performance testing of the polypropylene material, the displacement rate was 3 mm/min.

## Supplementary Material

pgad098_Supplementary_Data

## Data Availability

All research data are included in the article and/or supporting information.
