# Supplementary material for: Multi-triangles cylindrical origami and inspired metamaterials with tunable stiffness and stretchable robotic arm
Source: PNAS Nexus. 2023 Mar 23;2(4):pgad098. doi: 10.1093/pnasnexus/pgad098 (PMC10096905; doi:10.1093/pnasnexus/pgad098)
Supplement: pgad098_Supplementary_Data [file pgad098_supplementary_data.zip › PNASNEXUS-PNASNEXUS-2022-01230-T-s08.pdf]

Supporting Information for

Multi-triangles cylindrical origami and inspired metamaterials with tunable stiffness and stretchable robotic arm

Xiaolei Wang, Haibo Qu, Xiao Li, Yili Kuang, Haoqian Wang, and Sheng Guo

Haibo Qu and Sheng Guo.
E-mail: hbqu@bjtu.edu.cn and shguo@bjtu.edu.cn

This PDF file includes:

Supporting text
Figs. S1 to S20
Tables S1 to S5
Legends for Movies S1 to S7
SI References

Other supporting materials for this manuscript include the following:

Movies S1 to S7

Supporting Information Text

Note 1: Geometry of the multi-triangles cylindrical origami

The flat sheet and geometric parameters of the MTCO during the folding motion are shown in Fig. S2. According to the sine and cosine theorems of triangles

$$L_{AB} = a \quad [1]$$

$$L_{AD} = a\frac{\sin\alpha}{\sin\beta} \quad [2]$$

$$L_{BD} = a\frac{\sin(\alpha+\beta)}{\sin\beta} \quad [3]$$

$$L_{AC} = \sqrt{L_{AB}^2 + L_{BC}^2 - 2L_{AB}L_{BC}\cos(\alpha+\beta)} = a\sqrt{1+\frac{\sin^2\alpha}{\sin^2\beta} - 2\frac{\sin\alpha}{\sin\beta}\cos(\alpha+\beta)} \quad [4]$$

The coordinates of each point are given by constructing a coordinate system, where E_1 is the midpoint of the valley crease line BD, and E_3 is the midpoint of the mountain crease line AC.

$$O(0,0,0) \quad A\left(R\cos\left(\frac{2\pi}{n}\right), -R\sin\left(\frac{2\pi}{n}\right), 0\right) \quad B(R,0,0)$$

$$C(R\cos\varphi, -\mathrm{R}\sin\varphi, h) \quad \mathrm{D}\left(\mathrm{R}\cos\left(\frac{2\pi}{n}+\varphi\right), -R\sin\left(\frac{2\pi}{n}+\varphi\right), h\right)$$

$$E_1\left(\frac{R}{2}\left(\cos\left(\frac{2\pi}{n}+\varphi\right)+1\right), -\frac{R}{2}\sin\left(\frac{2\pi}{n}+\varphi\right), \frac{h}{2}\right)$$

$$E_3\left(\frac{R}{2}\left(\cos\left(\frac{2\pi}{n}\right)+\cos\varphi\right), -\frac{R}{2}\left(\sin\left(\frac{2\pi}{n}\right)+\sin\varphi\right), \frac{h}{2}\right)$$

$$E_2\left(mE_1(X)+(1-m)E_3(X), mE_1(Y)+(1-m)E_3(Y), \frac{h}{2}\right)$$

The structural form of the MTCO at the first and second stable states is that the unit cell is divided into two triangles by the valley crease line BD. However, the unit cell is divided into two triangles by the mountain crease AC at the third stable state. Therefore, during the MTCO is folded from the first stable state to the second one (the first folding process), the unit cell is divided into two triangular surfaces ΔABD and ΔBCD by the valley crease line BD. During the MTCO is folded from the second stable state to the third one (the second folding process), the two triangular surfaces are again divided by the mountain crease line AC, which is expressed as four triangular surfaces ΔABE_2, ΔBCE_2, ΔCDE_2, and ΔADE_2. During the second folding process, intersection E_2 of crease lines BD and AC is located between the midpoint of the valley crease line BD and midpoint of the mountain crease line AC, and it is a dynamic process. At the beginning of the second folding process, point E_2 coincides with E_1. At the end of the second folding process, point E_2 coincides with E_3. We introduce m $(0 \le m \le 1)$ here and use the sum of E_1 and E_3 to represent the coordinate of the point E_2. Because the MTCO follows the minimum energy path during the folding motion, the corresponding value of m can be found in the solving process of MATLAB.

Then, the length of each crease line can be expressed as

$$l_{AB} = 2R\sin\left(\frac{\pi}{n}\right) \quad [5]$$

$$L_{AD} = \sqrt{h^2 - 2R^2\cos\varphi + 2R^2} \quad [6]$$

During the first folding process

$$l_{BD\_1} = \sqrt{h^2 - 2R^2\cos\left(\frac{2\pi}{n}+\varphi\right) + 2R^2} \quad [7]$$

$$l_{AC\_1} = l_{AE_1} + l_{CE_1} \quad [8]$$

During the second folding process

$$l_{BD\_2} = l_{BE_2} + l_{DE_2} \quad [9]$$

$$l_{AC\_2} = l_{AE_2} + l_{CE_2} \quad [10]$$

The subscript _1 and _2 represent the first and second folding processes respectively.

Note 2: The modified truss model

We should keep in mind that the truss model is used only for its ability to provide a qualitative understanding of the general behavior of Kresling pattern origami, and as a guideline for the choice of the design parameters that lead to qualitatively different properties. Many reasons have been mentioned by Masana (1), the truss model cannot be used as a means for quantitative prediction of the behavior of Kresling pattern origami. Using the truss model, the energy change during the folding motion can be obtained. However, with the development of research on Kresling pattern origami, we find that part of the energy change obtained from the truss model is not consistent with the actual folding motion of Kresling pattern origami. The main reason for this problem is that, during the folding process from the second stable state position, the valley crease lines BD gradually take on an arc shape. At this time, there is an error in calculating the lengths of the crease lines using space coordinate points, which also causes the energy obtained from the truss model to increase sharply after the second stable state position. In fact, there is a stable state during the late folding motion of the MTCO. The main feature is that the MTCO cell is divided into two triangular planes by the mountain crease line AC, as shown in Fig. S3. However, the energy landscape obtained by the truss model does not capture this property.

Based on the characteristics of the structural changes, the folding process of the MTCO is represented in the truss model as two stages, as shown in Table S1 & S2. The essence of the truss model is to calculate the strain energy caused by stretching or shortening the crease lines during the folding process, and the crease lines in the MTCO cell are AD, BD, and AC. Therefore, during the first folding process, the structural form of the two triangular panels is represented by the crease lines AD and BD, and the crease line AC is divided by BD. Point E_1 is the midpoint of crease line BD, and the coordinates of point E_1 can be obtained directly. Then the mountain crease line AC can be represented by AE_1 and CE_1, and the equation of deformation energy can be expressed as:

$$U = \frac{1}{2}k(\Delta L_{AD}^2 + \Delta L_{BD}^2 + \Delta L_{AE_1}^2 + \Delta L_{CE_1}^2) \qquad [11]$$

where, $\Delta L_{AD} = l_{AD} - L_{AD}$, $\Delta L_{BD} = l_{BD} - L_{BD}$, $\Delta L_{AE_1} = l_{AE_1} - 1/2L_{AC}$, $\Delta L_{CE_1} = l_{CE_1} - 1/2L_{AC}$.

During the second folding process, the crease lines AC and BD are divided by each other, and the structural form of the four triangular surfaces (ΔABE_2, ΔBCE_2, ΔCDE_2, and ΔADE_2) is represented by the crease lines AE_2, BE_2, CE_2, and DE_2. Point E_2 is a moving point between the midpoint of crease line BD and the midpoint of crease line AC. The valley crease line BD is represented by BE_2 and DE_2, and the mountain crease line AC is represented by AE_2 and CE_2. The equation can be expressed as:

$$U = \frac{1}{2}k(\Delta L_{AD}^2 + \Delta L_{BE_2}^2 + \Delta L_{DE_2}^2 + \Delta L_{AE_2}^2 + \Delta L_{CE_2}^2) \qquad [12]$$

where, $\Delta L_{AD} = l_{AD} - L_{AD}$, $\Delta L_{BE_2} = l_{BE_2} - 1/2L_{BD}$, $\Delta L_{DE_2} = l_{DE_2} - 1/2L_{BD}$, $\Delta L_{AE_2} = l_{AE_2} - 1/2L_{AC}$, $\Delta L_{CE_2} = l_{CE_2} - 1/2L_{AC}$.

The top and bottom polygons are assumed as rigid, and the deformation energy U depends on the folding height h and torsion angle φ. The energy landscape and minimum energy path can be obtained by applying $\partial U/\partial\varphi = 0$. The modified truss model is not only suitable for the MTCO proposed, but also validates the problem that existed in the general truss model for the triangulated conical origami (2) and triangulated cylindrical origami (3, 4). From the energy landscape obtained by the modified truss model, it can be observed clearly that there are three energy extreme points during the folding process ($\alpha = 38°, \beta = 30°$), as shown in Fig. 2$C$.

The MTCO can not be stretched further when it is at the third stable state, and the triangulated conical origami and triangulated cylindrical origami exhibit the same phenomenon, meaning that there is an ending position for the folding motion of Kresling pattern origami. Therefore, the boundary condition for the truss model is given as $L_{AC} = l_{AC\_2}$. Compared to the previous truss model, this boundary condition is also part of the model correction.

In Fig. S4, an obvious mutation can be observed in the relationship between the torsion angle and folding height obtained using the modified truss model. This position is the boundary point of the two folding processes and the second stable state position. In the previous truss model, the energy increases sharply within a small height range from this position, which is also the fundamental reason for ignoring the third stable state of Kresling pattern origami. This energy mutation is generated by the structural changes of the MTCO during the folding process, resulting in a different calculation method for the crease lines expressed in the truss model.

The samples of the MTCO and origami-inspired metamaterial ($\alpha = 38°$, $\beta = 30°$) were compressed to validate the energy landscape obtained from the modified truss model, and the load/displacement curves are shown in Fig. S5. The load/displacement curves obtained from the compression experiments represent the folding process from the third stable state to the first one, while the energy landscape obtained from the truss model is the folding process from the first stable state to the third one, and thus is reversed on the horizontal axis. The load/displacement curves are divided into four stages, the first stage represents the load-bearing process at the third stable state, the second stage shows the folding process from the third stable state to the second one, and the third and fourth stages denote the folding process from the second steady state to the first one. According to the blue part of the experimental curves (the second stage), the folding process of the samples from the third to the second stable state is also an obvious mutation, which is consistent with the calculation results of the modified truss model. The fluctuations of the experimental curves are due to the errors formed by the hand-made samples

and the vibration of the environment, which cause multiple unit cells of the samples to be compressed gradually rather than simultaneously (as shown in Fig. S5, the compression process between ① and ③). In the truss model, the top and bottom polygons are assumed horizontal, implying that multiple unit cells are compressed simultaneously. Thus, the energy landscape shows a fluctuation-free mutation. In addition, the first stable state of the actual samples does not occur when the height is close to 0, but this stable state does exist, which is consistent with the results of the multi-state analysis of the truss model. The reasons for this situation are that the truss model neglects the thickness, material properties, and nonlinearities during the folding process. In the truss model, the energy jump is caused by a change in the calculation method of the crease lines. In the compression experiments, the mutation in the force is due to the folding of the unit cells. In general, an unusual mutation in energy arises because of the change in the structural form of the MTCO during the second folding process.

Note 3: Categories for the multi-triangles cylindrical origami

Based on whether the panels need to be involved in deformation during the folding process, it can be classified into rigid and non-rigid origami. A typical example of rigid origami is the Miura pattern origami with a single DOF, and the original single-layer Miura origami can only be folded as a plane or shell. As research progressed, different mounting methods were designed to investigate the negative Poisson's ratio, adjustable stiffness, and stability properties (5–7). Compared to the Kresling pattern origami, the panel of the Yoshimura pattern origami undergoes large deformation during the folding process, resulting in very complex nonlinear problems (8, 9). Kresling pattern origami requires only appropriate panel deformation during the folding process, while bistable properties can be achieved according to adjustable geometric parameters. Traditional Kresling pattern origami mainly includes triangulated cylindrical origami and triangulated conical origami, which are widely observed for their bistable property (2, 10, 11). The multi-triangles cylindrical origami with tristable property is defined as a new structure of Kresling pattern origami, which provides a larger range of stiffness adjustment and richer motion patterns than traditional triangulated cylindrical origami, creating new content for the research of Kresling pattern origami.

MTCO is classed into four cases according to the energy landscape obtained from the modified truss model and the deployable characteristics. First, three stable states of the MTCO are defined, as shown in Fig. S3 represents the first stable state, second stable state, and third stable state respectively.

Case1: The characteristic of this case is that the MTCO has the second and third stable states, and the energy barrier between the two states is much lower than the one before the second stable state. At this point, the MTCO can be folded between the second and third stable states. If the MTCO is folded from the second stable state to a compact planar state, the panels and creases produce strain considerably, causing irreparable panel damage. This case is called bistable locally deployable origami. An example is $\alpha = \beta = 35°$, the energy landscape of which is shown in Fig. S4A.

Case2: The characteristic of this case is that the MTCO has the second and third stable states, and the energy barrier between the two states is much higher than that before the second stable state. At this point, the MTCO can be folded to a compact planar state. This case is called bistable fully deployable origami. An example is $\alpha = \beta = 32°$, the energy landscape is shown in Fig. S4B. From the perspective of the energy landscape, the difference between this case and the case1 is that the amount of energy between the second and third stable states is different from the one before the second stable state.

Case3: This kind of the MTCO has three stable states. The energy barrier between the second and third stable states is higher than the one between the first and second stable states. The MTCO can be folded during the whole process. This case is called tristable fully deployable origami. Typical example is $\alpha = 38°$ and $\beta = 30°$, and its energy landscape is shown in Fig. S4C. This case is the research hotspot of Kresling pattern origami.

Case4: This type of the MTCO has zero stiffness and the third stable state properties, which is a special case of Kresling pattern origami. The MTCO can be folded to a compact planar state, which is called zero stiffness fully deployable origami. An example is $\alpha = \beta = 30°$, whose energy landscape is shown in Fig. S4D.

The MTCO with $\alpha + \beta \geq 90°$ has the second and third stable states, which can be folded between the two stable states. Therefore, this is one of the Case1. However, in both the previous and modified truss models, the obtained relationships between the torsion angle and folding height show that the torsion angle remains constant and zero between the second and third stable states, which is not consistent with the actual folding process. Meanwhile, the two truss models represent different stable state positions. The previous truss model shows the second stable state (Fig. S6A), whereas the modified truss model shows the third stable state (Fig. S6B). The MTCO with $\alpha + \beta \geq 90°$ is extremely difficult to be compressed from the second stable state to planar state, and the minimum energy path represented by the truss model must to be validated further. The load-bearing capacity experiments on the MTCO samples (Case1) were carried out to validate the applicability of the truss model, as shown in Fig. S7. For the MTCO with $\alpha = \beta = 35°$, the weight that the MTCO sample could carry at the second stable state was 2 Kg, and the consequences was 6 Kg at the third stable state, as shown in Fig. S7A. Similarly, the weight that the sample of the MTCO with $\alpha = \beta = 50°$ could carry at the second stable state was 4 Kg, and the consequences was 6 Kg at the third stable state, as shown in Fig. S7B. Both experimental results show that the MTCO (Case1) has a higher stiffness at the third stable state relative to the second one. However, the truss model exhibits an energy barrier opposite to the experimental results, meaning that the minimum energy path is problematic. It can be concluded that the truss model is not applicable to the MTCO when there are two stable states and the truss model expresses a higher stiffness of the second stable state relative to the third one.

Note 4: The tristable property of Kresling pattern origami

The structural forms of Kresling pattern origami mainly include the triangulated conical form and triangulated cylindrical form. Of course, multi-triangles cylindrical form proposed here is also included. Previous research emphasize that Kresling pattern origami has bistable property, while ignoring the third stable state. Essentially, the third stable state exists not only in the multi-triangles cylindrical origami but also in the triangulated conical origami and triangulated cylindrical origami.

For the third stable state of the MTCO, the main feature is that the MTCO cell is divided into two triangular planes by mountain crease line. For the triangulated cylindrical origami and triangulated conical origami, the main feature is that each unit cell presents an outward-bending quadrilateral surface. Previous research has shown structural features similar to the third stable state of Kresling pattern origami, but this property has not been pointed out. For example, in the research by Yasuda (4), the structural feature of the third stable state is demonstrated, but it is to express the origami characteristics corresponding to the origami-inspired structure at a certain time.

In general, previous research has not revealed the third stable state of Kresling pattern origami. In this paper, the third stable state of Kresling pattern origami is identified by proposing the MTCO and modifying the truss model, meaning that Kresling pattern origami has tristable property, not just bistable property.

Note 5: The phenomenon of the changed definition of the crease lines

There is a phenomenon that needs to be noticed, the definition of the crease lines expressed at the second and third stable states of the MTCO ($\alpha + \beta > 90°$) does not correspond to the one expressed in the flat sheet. For the flat sheet of the MTCO ($\alpha = \beta = 50°$), AC is defined as a mountain crease line, and BD is defined as a valley crease line, as shown in Fig. S8*A*. At the second and third stable states of the MTCO, AC becomes a valley crease line, and BD becomes a mountain crease line, as shown in Fig. S8*B*. This situation is the same as Kresling pattern origami, which has been shown in Zhai's research (3). It can be observed that Kresling pattern origami exhibits different definition of crease lines from that in the flat sheet when the geometric parameters are $\alpha + \beta > 90°$.

We consider $\alpha + \beta = 90°$ as a condition for dividing the MTCO into two different forms. One form is that the definition of the crease lines does not change between the structural expression and flat sheet design ($\alpha + \beta < 90°$), and a flat sheet with oppposite chirality corresponding to the flat pattern cannot be found. The other form is that the definition of the crease lines is changed between the structural expression and flat sheet design ($\alpha + \beta > 90°$). In this case, a corresponding flat sheet with opposite chirality can be found, as shown in Fig. S8*C*. These two flat sheets are identical in the structural form of MTCO. However, regardless of the expression of the flat sheets, the corresponding MTCO belongs to the Case1, so the truss model is still not applicable (*SI Note* 3). Therefore, when the geometric parameter is $\alpha + \beta > 90°$, it is more accurate to find the corresponding flat sheet with opposite chirality to express the MTCO structure.

Note 6: The point-searching method for the special stable states

Research on dynamics of Kresling pattern origami is mainly based on the truss model of the finite element folding structure. An important assumption is that the top polygon shares a rotation axis with bottom polygon. That is a coupled degree of freedom, meaning that the top and bottom polygons are always parallel. Although there are more complex folding mechanisms during the folding process, the truss model is convincing to analyze the energy changes and stable state forms of Kresling pattern origami. However, the special stable states mentioned in this study (as shown in Fig. 3*C*) are mainly characterized by the fact that the structural forms of the second and third stable states exist in the unit cells at the same time, which causes a large amount of deformation in the top and bottom polygons, and they are not parallel. Therefore, the truss models can not be used to analyze these special stable states.

Here, we utilize a point-searching method to explain these special stable states during the folding process of the MTCO. This method still assumes that the elastic energy of the MTCO is caused by stretching or shortening the crease lines in the unit cells. Subsequently, 12 vertices of the MTCO (n=6) are searched in a spatial coordinate system to satisfy that the elastic energy caused by the crease lines is a local minimum point. The coordinates of the 12 vertices of the MTCO can be obtained, forming the structures of these stable states.

First, 12 vertices are used to describe the MTCO. The vertices of the bottom polygon are A_1, A_2, …, A_6, and those of the top polygon are B_1, B_2, …, B_6. Then, the MTCO can be represented by 24 crease lines, A_1A_2, A_2A_3, …, A_6A_1, B_1B_2, B_2B_3, …, B_6B_1, A_1B_1, A_2B_2, …, A_6B_6 and the active crease lines within the 6 unit cells (such as valley crease line A_2B_1 or mountain crease line A_1B_2). When the MTCO is at a special stable state, the elastic energy within the structure can be expressed as

$$U = \frac{1}{2}k\sum_{i=1}^{6}(\Delta L^2_{A_iA_{i+1}} + \Delta L^2_{B_iB_{i+1}} + \Delta L^2_{A_iB_i}) + i\Delta L^2_{A_iB_{i+1}} + (6-i)\Delta L^2_{A_{i+1}B_i} \quad [13]$$

where, A_7 is the same point as A_1, B_7 is the same point as B_1, i represents the number of the form of the third stable state in the unit cells (i=1, 2,…, 6), and k is the elastic constant of the structure. $\Delta L_{A_iA_{i+1}} = l_{A_iA_{i+1}} - L_{A_iA_{i+1}}$ is the deformation of the crease line A_iA_{i+1}, $\Delta L_{B_iB_{i+1}} = l_{B_iB_{i+1}} - L_{B_iB_{i+1}}$ is the deformation of the crease line B_iB_{i+1}, $\Delta L_{A_iB_i} = l_{A_iB_i} - L_{A_iB_i}$ is the deformation of the crease line A_iB_i, $\Delta L_{A_iB_{i+1}} = l_{A_iB_{i+1}} - L_{A_iB_{i+1}}$ is the deformation of the crease line A_iB_{i+1}, and

$\Delta L_{A_{i+1}B_i} = l_{A_{i+1}B_i} - L_{A_{i+1}B_i}$ is the deformation of the crease line $A_{i+1}B_i$. $l_{A_iA_{i+1}}$ represents the distance of coordinate point A_i and A_{i+1}, $L_{A_iA_{i+1}}$ is the length of the crease line A_iA_{i+1} at the planar state, and the rest of the expressions are the same.

Therefore, expressing the special stable states of the MTCO during the folding process is transformed into a mathematical problem: in a global coordinate system, given an initial point (as shown in Fig. S9A), searching the 11 coordinate points corresponding to the minimum of the elastic energy expression (U/k) of the origami structure, and satisfying that the deformation of each crease line is within 2% (3, 11). In other words, under the 24 inequality boundary conditions (corresponding to the 24 crease lines), searching the minimum of the single objective function (U/k) and the corresponding coordinate points. MATLAB is used to solve this mathematical problem, and the results are shown in Fig. S9$B - F$ and Table $S3 - S5$ (a=30 mm).

The idea of the point-searching method is the same as that of the truss model, which simply considers that the elastic energy of Kresling pattern origami is caused by stretching or shortening the crease lines. However, the point-searching method ignores the deformation stress within the origami material, and the results show relatively large fluctuations. The result of the second stable state obtained from the point-searching method is in agreement with that obtained from the truss model, validating the accuracy of the point-searching method, as shown in Table $S3$ and Fig. S9B. Meanwhile, the results of the third stable state obtained by the point-searching method and the truss model are compared to validate the accuracy of the modified truss model again, as shown in Table $S4$ and Fig. S9C. Using the point-searching method, the structural forms of the MTCO with special stable states (i=3) are obtained, as shown in Table $S5$ and Fig. S9$D - F$.

Note 7: Materials and Methods

The experimental origami samples were made with 200 g/m^2 or 120 g/m^2 cardboard. The flat sheet of Kresling pattern origami was printed on cardboard, and the pattern was cut down along the outer contour. Then the cardboard was folded according to the definition of the crease lines. Finally, the ends of the cardboard were glued to the top/bottom polygon surface with double-sided tape to form a closed shape. Cardboard with 200 g/m^2 has better stiffness, which was used for stiffness analysis and characteristic expression. However, cardboard with 120 g/m^2 is easier to fold, which was used to produce the origami robotic arm.

The origami-inspired metamaterials were made of polypropylene sheets with 0.5 mm thick. A laser engraving machine was used to cut the polypropylene sheet along the crease lines to form the flat pattern, as shown in Fig. S10B. Then the pattern was folded as defined by the crease lines, and the ends were glued with double-sided tape. The top/bottom polygon panels were fabricated using 3D printing. The polygon panels are different for various metamaterials, as shown in Fig. S10C. Finally, the single-module metamaterial was completed by attaching the folded polypropylene sheet to polygon plates with M1.4*5 mm bolts. A Plexiglas plate was used to connect multiple single-module metamaterials to create modular metamaterials. Flat bearings were installed at the top and bottom to ensure rotational DOF of the modular metamaterials. Photos of the metamaterial samples are shown in Fig. S11.

The origami-inspired robotic arm was made of 120 g/m^2 cardboard, and a film was applied to the outer surface of the cardboard to avoid damaging the cardboard too much after repeated usage, as shown in Fig. S17. The film is typically used for the fabrication of airplane models and can be directly attached to the outer surface of the cardboard after heating. The cardboard covered with the film was folded according to the definition of the crease lines and then glued with double-sided tape to form the origami robotic arm. The robotic arm used for stiffness testing (Fig. 5B & C) was connected between the single-module origami units with polygonal Plexiglas plates. The rest of the robotic arms were fabricated by directly connecting single-module origami units. For the origami robot arm, the top and bottom polygons used for connecting were cut down, which allows the robotic arm to be controlled more easily.

Note 8: Performance measurements of the polypropylene material

To investigate the mechanical properties of the polypropylene material used for the metamaterials, the tensile experiments were carried out with a universal tensile testing machine. The tensile rate during the experiments was 3mm/min. The designed sample size was 115 mm * 10 mm * 0.5 mm($L * b * h$), as shown in Fig. S12A. The curves of the test data are shown in Fig. S12B.

According to Hooke's law, within the elastic limit of an object, the stress of the object is proportional to its strain.

$$\frac{F}{A} = Y\frac{\Delta L}{L} \qquad [14]$$

The Young's modulus can be expressed as

$$Y = \frac{FL}{A\Delta L} \qquad [15]$$

where A is the cross-sectional area of the sample. Taking the data points within the elastic stretching in the experiments, the Young's modulus of the polypropylene material obtained was 869.6 Mpa.

Poisson's ratio refers to the absolute ratio of the transverse normal strain to the axial normal strain of the material under uniaxial tension or compression, also known as the transverse deformation coefficient. It is an elastic constant that reflects the

transverse deformation of the material.

$$\mu = -\frac{\varepsilon_x}{\varepsilon_y} \tag{16}$$

In the elastic deformation range of the material, the transverse deformation of the material corresponding to the axial deformation was measured, and Poisson's ratio of polypropylene material calculated was about 0.345.

In a tensile experiment, the maximum tensile stress that the material subjected can be expressed as

$$\sigma = \frac{F_{max}}{A} \tag{17}$$

Taking the maximum force point in the experiments, the calculated tensile strength of the polypropylene material was 22.36 Mpa.

The elongation is the ratio of the fracture length to the initial length, which can be expressed as

$$\delta = \frac{\Delta L}{L} * 100\% \tag{18}$$

Taking the length of the experimental breaking point, the elongation of the polypropylene material calculated is about 8%.

Note 9: Compression experiments and data analysis of the metamaterials

Origami patterns are essentially metamaterials. However, the effects of different material properties on the origami properties are different. To utilize the high stiffness property of the third stable state, while retaining multiple stable states of Kresling pattern origami, polypropylene sheets with 0.5 mm thick were applied to create the metamaterials. The metamaterials have deployable, multi-stable, and tunable stiffness properties (Movie S6). The tunable stiffness of the metamaterials is inspired by the high stiffness of the third stable state. Therefore, the mechanism design to maintain the third stable state is an idea to improve the stiffness of the metamaterial.

A universal tensile testing machine was used to test the load-bearing capacity of the metamaterials. Similar to Kresling pattern origami, the metamaterials have a coupled DOF of rotation and compression. If the single-metamaterial was placed directly in the middle of the fixtures, the friction could constrain its rotation, causing the sample not to be folded following the folding path. Therefore, flat bearings were placed between the fixtures to ensure rotational DOF of the metamaterial. Three load/displacement curves show that the load-bearing capacity of the metamaterial did not decrease significantly after multiple compressions, which is an advantage of polypropylene material. By arranging reversed magnets and springs inside the metamaterial, the third stable state can be maintained, while maintaining deployable property. The load/displacement curves obtained show that the peak load of the metamaterials with magnets and springs inside was significantly increased.

The tristable property and high stiffness property of the third stable state of the MTCO are extended to the traditional Kresling pattern origami, implying that the TCO also has these properties. To illustrate these properties, a TCO-inspired metamaterial composed of polypropylene is designed ($\alpha = 38°, \beta = 30°$). In contrast to two stable triangular panels at the third stable state of the MTCO, the TCO unit cell exhibits an outwardly curved surface. Therefore, the TCO has relatively poor stability and is more likely to be compressed along the folding path. The TCO-inspired metamaterial was intermittently folded back to the second stable state during the compression process, and the load/displacement curve exhibited a large fluctuation, as shown in Fig. S13C. Meanwhile, compared with the MTCO-inspired metamaterial, the load-bearing capacity of the TCO-inspired metamaterial at the third stable state was relatively low, which is also caused by the instability induced by the intermittent folding of the unit cells.

The modular design concept allows for different spatial dimensions and increases the load-bearing capacity of the single-module metamaterial. Of course, the modular arrangement of metamaterials will become less easy to be folded at the high stiffness stable state, which needs to be operated for each unit. The smaller diameter of the tensile machine fixtures results in a relatively concentrated pressure on the middle position. Therefore, during the compression of the modular metamaterials, the individual metamaterial located in the middle was folded first, after which the load-bearing capacity continued to increase. The load/displacement curves obtained show that when the middle single-module was compressed, the metamaterial with opposite chirality had a larger displacement (Fig. 4 *D* & *E*), meaning that the metamaterials with opposite chirality are more stable. To accurately illustrate this feature, four modular metamaterials were compressed, as shown in Fig. S14*C* & *E*. The load/displacement curves show that the opposite chirality arrangement results in higher load-bearing capacity and stability.

Note 10: Analysis on the motion features of the multi-triangles cylindrical origami

Kresling pattern origami always shares a common rotation axis during the folding motion, which is an important basis for investigating the truss model, meaning that the top and bottom polygons are always parallel. However, if external forces are applied to the unit cell, Kresling pattern origami can achieve both bending and twisting motions. Wu (12) combined Kresling pattern origami with bistable property and magnetically responsive materials to design a robotic arm. The robotic arm achieves omnidirectional twisting and bending, demonstrating that Kresling pattern origami has massive potential in fields such as robotic arms and robots. Thus, with the proposed third stable state of Kresling pattern origami, its stretchable range and bending degree are expanded again. Here, the motion features of the MTCO are analyzed in conjunction with the energy landscape obtained from the truss model.

Different geometric parameters correspond to different physical properties. Therefore, the change degree of their kinematic features is also different. Taking the MTCO with $\alpha = \beta = 30°$ as an example, the height corresponding to the end position of zero stiffness property (the second stable state position) is $h_2 = 0.51R$, and the height corresponding to the third stable state is $h_3 = 0.85R$, i.e., its stretchable range is expanded by 66.9%. If an external force is applied to a vertex of the MTCO so that it is in the form shown in Fig. S15E, it can achieve a bending angle of $\gamma_1 \approx 14.65°$. The bending angle could reach $\gamma_2 \approx 25.54°$, as shown in Fig. S15F. As a result, with the proposed tristable property of Kresling pattern origami, the bending degree is expanded by 74.3% ($(\gamma_2 - \gamma_1)/\gamma_1$). For the MTCO with $\alpha = \beta = 30°$, the torsion angle at the initial moment of zero stiffness is approximately 59.97°, meaning that point P coincides with point P'. Therefore, $\angle MPN$ can represents the angle between the top and bottom surfaces. However, for the MTCO with other parameters, the calculation of the angle between the top and bottom surfaces is lightly more complicated.

$$L_{MN} = L_{NP} = 2R \tag{19}$$

$$h_2 = 0.51R \tag{20}$$

$$h_3 = 0.85R \tag{21}$$

From the cosine theorem of triangles

$$\gamma_1 \approx 14.65° \tag{22}$$

$$\gamma_2 \approx 25.54° \tag{23}$$

When designing the robotic arm modularly, removing polygonal material connected between modules is conducive to deformation, and a larger motion range can be achieved under certain external drives. This makes the robotic arm exhibit the shape shown in Fig. S19, and the maximum bending angle of the independent unit could reach $\gamma_3 \approx 37.17°$. In this case, the connection between the origami units and the deformation of the end units is a huge challenge.

$$h' = L_{AC} = 1.275R \tag{24}$$

$$\gamma_3 \approx 37.17° \tag{25}$$

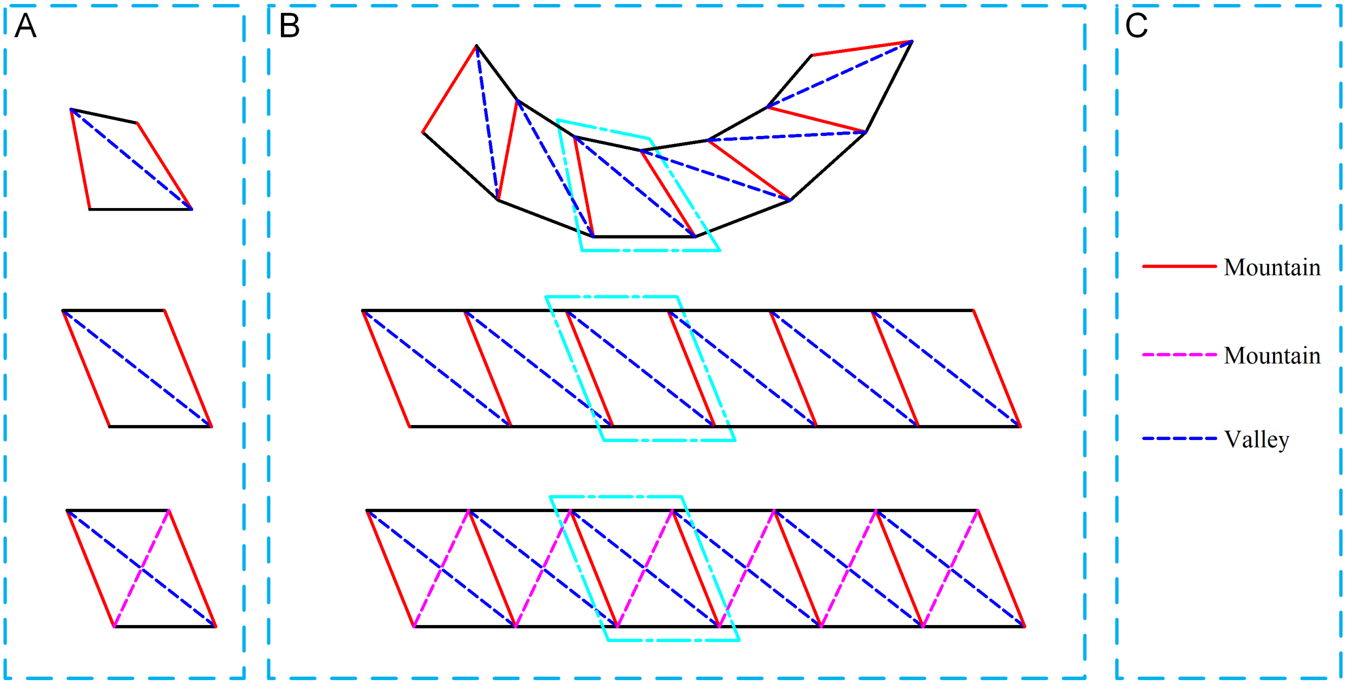

Fig. S1. The unit cells and flat sheets of Kresling pattern origami, including the triangulated conical origami, triangulated cylindrical origami and multi-triangles cylindrical origami. (A) The unit cells of Kresling pattern origami. (B) The flat sheets of Kresling pattern origami. (C) The definition of the crease lines of Kresling pattern origami.

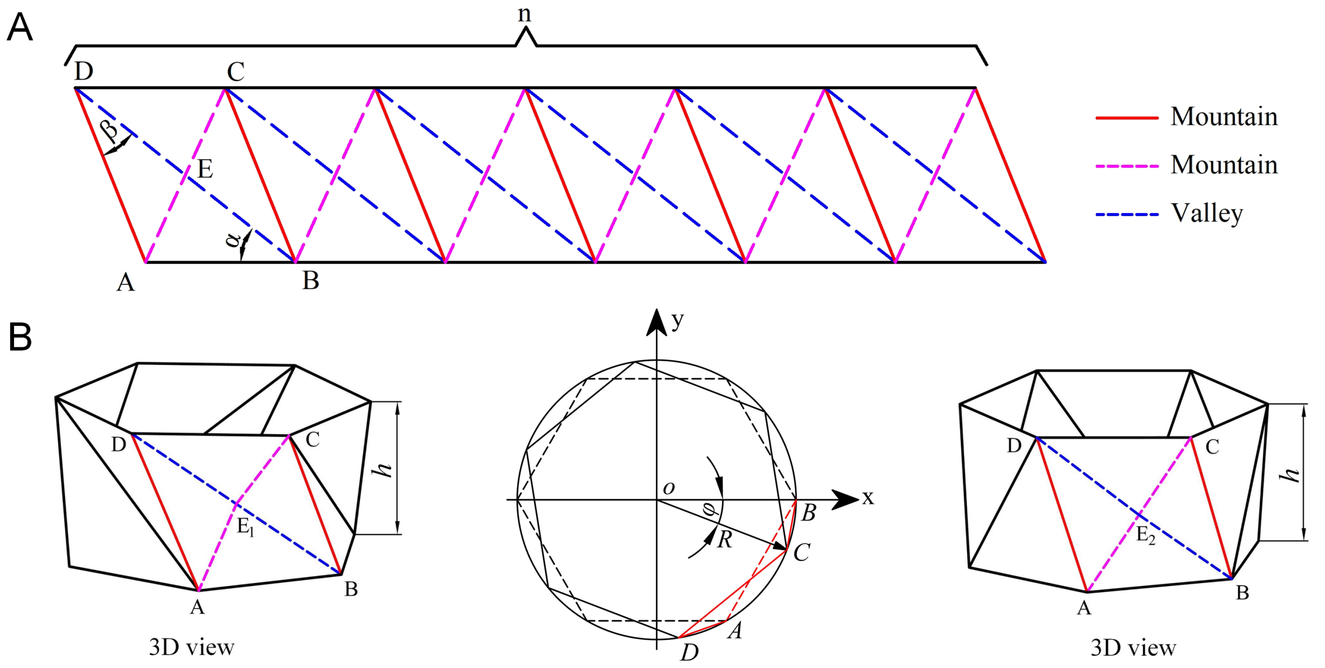

Fig. S2. Geometry of the multi-triangles cylindrical origami. (A) The flat sheet of the MTCO. (B) The geometrical parameters of the MTCO during the folding motion.

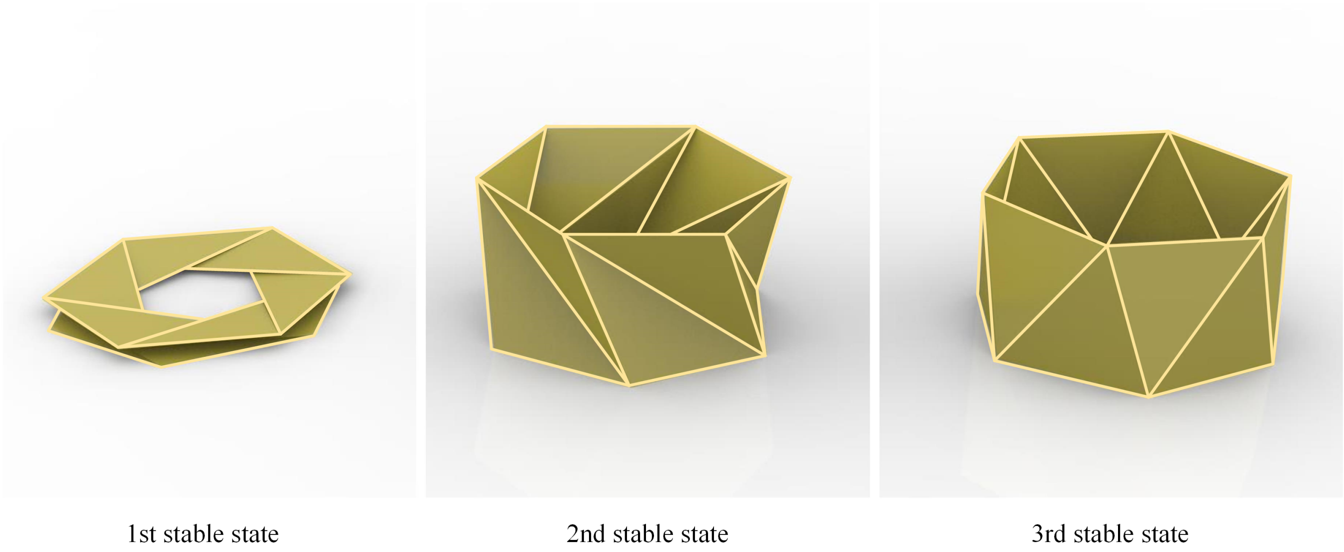

Fig. S3. Three stable states of the multi-triangles cylindrical origami. When stretching from the second stable state to third one, the unit cell of the multi-triangles cylindrical origami is divided into four triangular panels, which is a folding process. And when the multi-triangles cylindrical origami is at the third stable state, the unit cell is divided into two triangular panels.

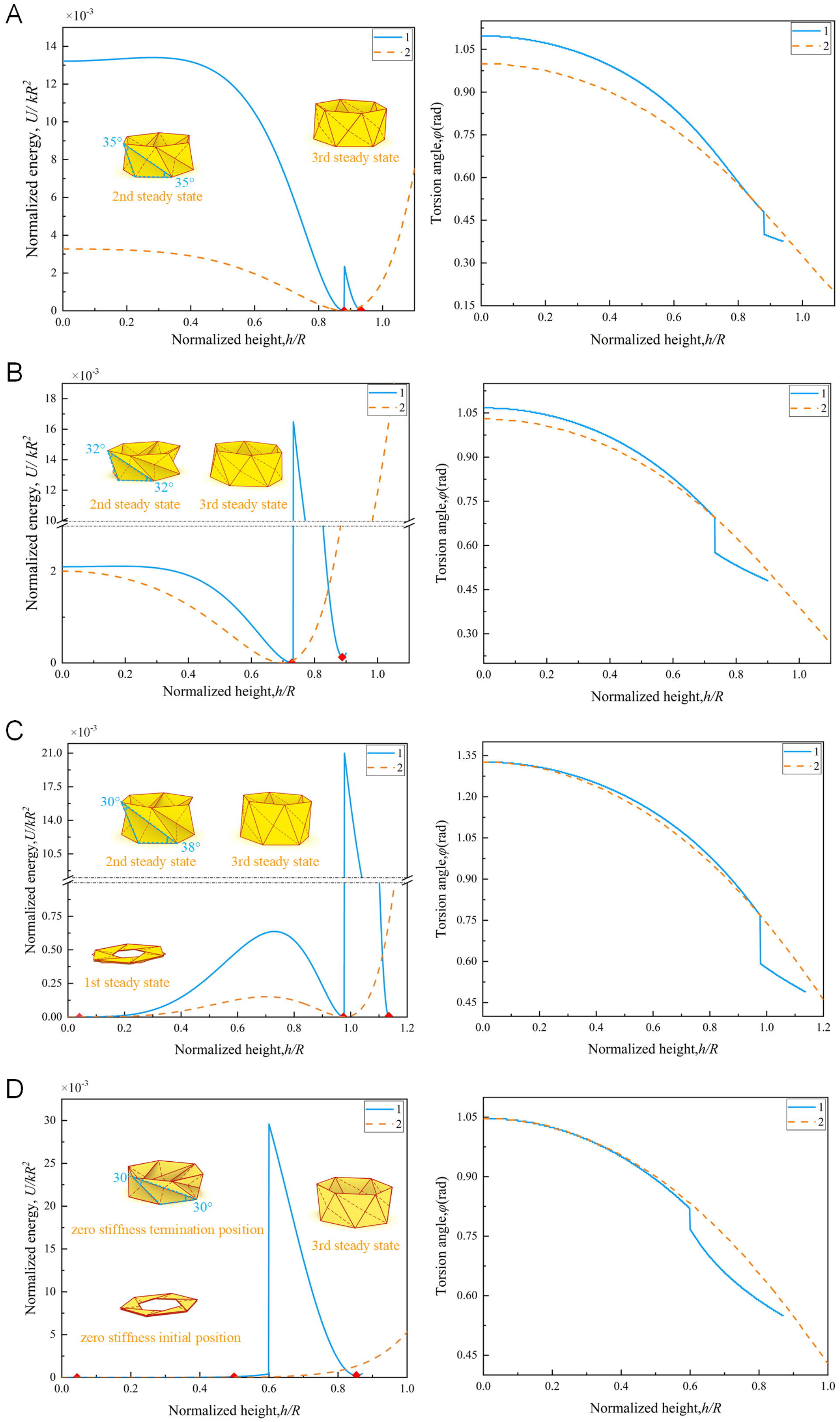

Fig. S4. The results obtained from the previous and modified truss models, including the energy landscapes and the relationships between the folding height and torsion angle. (A-D) The energy analysis for the MTCO-based truss model shows remarkably different behaviors: (A) Bistability at $\alpha = \beta = 35^\circ$. (B) Bistability at $\alpha = \beta = 32^\circ$. (C) Tristability at $\alpha = 38^\circ$, $\beta = 30^\circ$. (D) zero-stiffness mode at $\alpha = \beta = 30^\circ$.

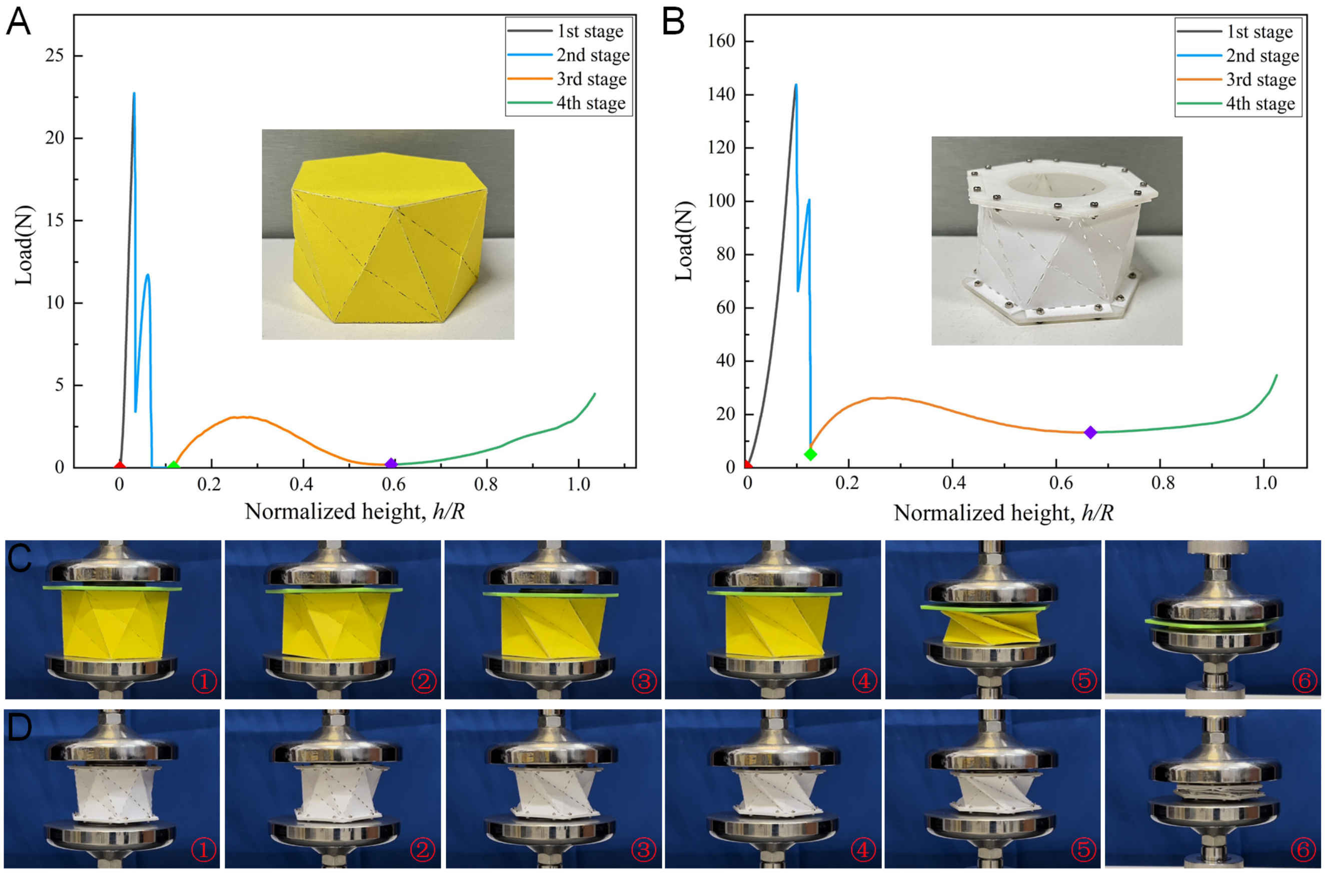

Fig. S5. The compression experiments for the samples of the multi-triangles cylindrical origami and origami-inspired metamaterial. (A) Load/displacement curve of the multi-triangles cylindrical origami sample. (B) Load/displacement curve of the origami-inspired metamaterial sample. (C) Structural features of the multi-triangles cylindrical origami sample during compression experiments. (D) Structural features of the origami-inspired metamaterial sample during compression experiments.

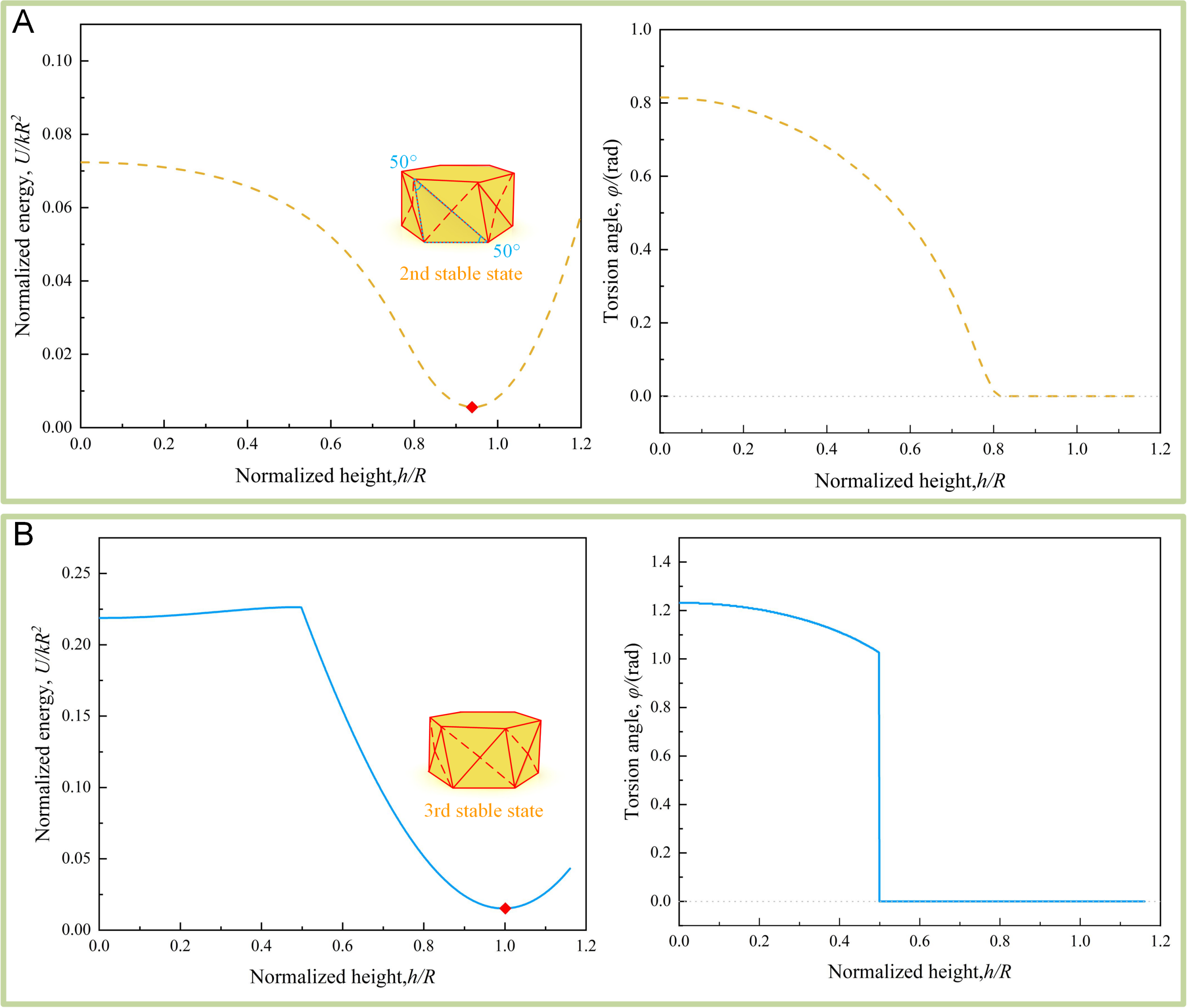

Fig. S6. Energy landscapes of the multi-triangles cylindrical origami with $\alpha = \beta = 50^\circ$. (A) Energy landscape obtained by the previous truss model. (B) Energy landscape obtained by the modified truss model. The torsion angle remains unchanged and zero in one phase. However, the MTCO with $\alpha = \beta = 50^\circ$ is deployable in the phase between the second stable state and the third one, but the torsion angle still remains unchanged, which is inconsistent with the actual folding motion.

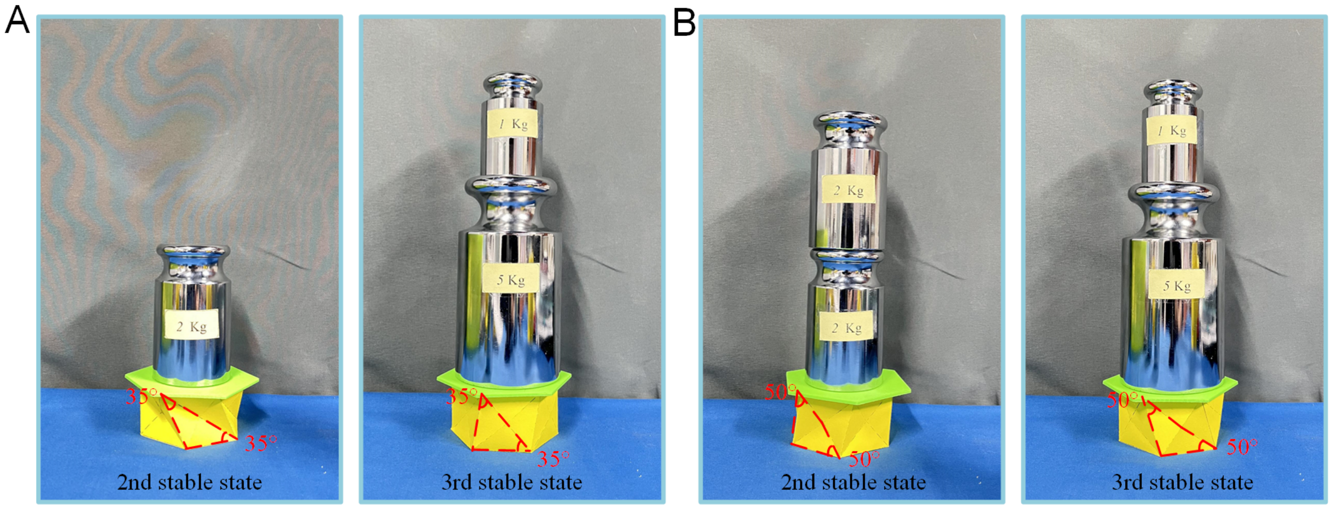

Fig. S7. Photos of the stiffness validated experiments. (A) For the MTCO with $\alpha = \beta = 35^{\circ}$, the weights that can be carried were 2 Kg at the second stable state and 6 Kg at the third stable state. (B) For the MTCO with $\alpha = \beta = 50^{\circ}$, the weights that can be carried were 4 Kg at the second stable state and 6 Kg at the third stable state. The results show that the MTCO (Case1) has higher stiffness at the third stable state compared to the second one, which also validates the applicability of the truss model.

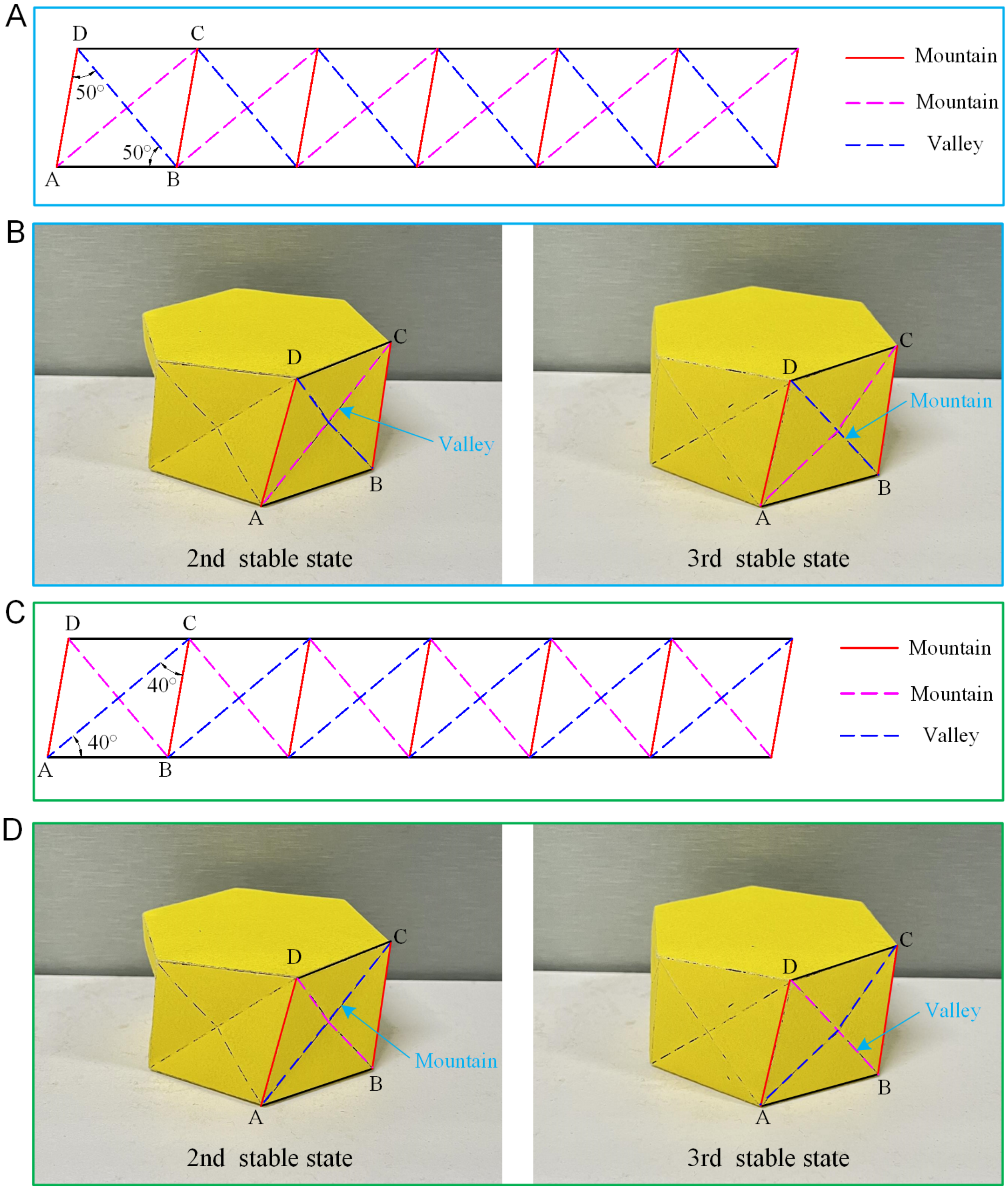

Fig. S8. Instruction on the changed definition of the crease lines of the multi-triangles cylindrical origami. (A) For the MTCO with $\alpha = \beta = 50°$, the definition of the crease lines is that AC is the mountain crease line and BD is the valley crease line. (B) Structural characteristics of the MTCO corresponding to the geometric parameters of $\alpha = \beta = 50°$, which expresses that AC is the valley crease line and BD is the mountain crease line, meaning that the the definition of the crease lines is changed. (C) For the MTCO with opposite chirality ($\alpha = \beta = 40°$), the definition of the crease lines is that AC is the valley crease line and BD is the mountain crease line. (D) Structural characteristics of the MTCO with opposite chirality corresponding to the geometric parameters of $\alpha = \beta = 40°$, which expresses that AC is the mountain crease line and BD is the valley crease line, meaning that the the definition of the crease lines is consistent with the flat sheet. For the corresponding flat sheet with opposite chirality, the position of the crease lines are the same at the plane state. However, the definition of crease lines is reversed, as shown in Fig. A & C. Therefore, when the geometric parameter is $\alpha + \beta > 90°$, it is more accurate to find the corresponding flat sheet with opposite chirality to express the MTCO structure.

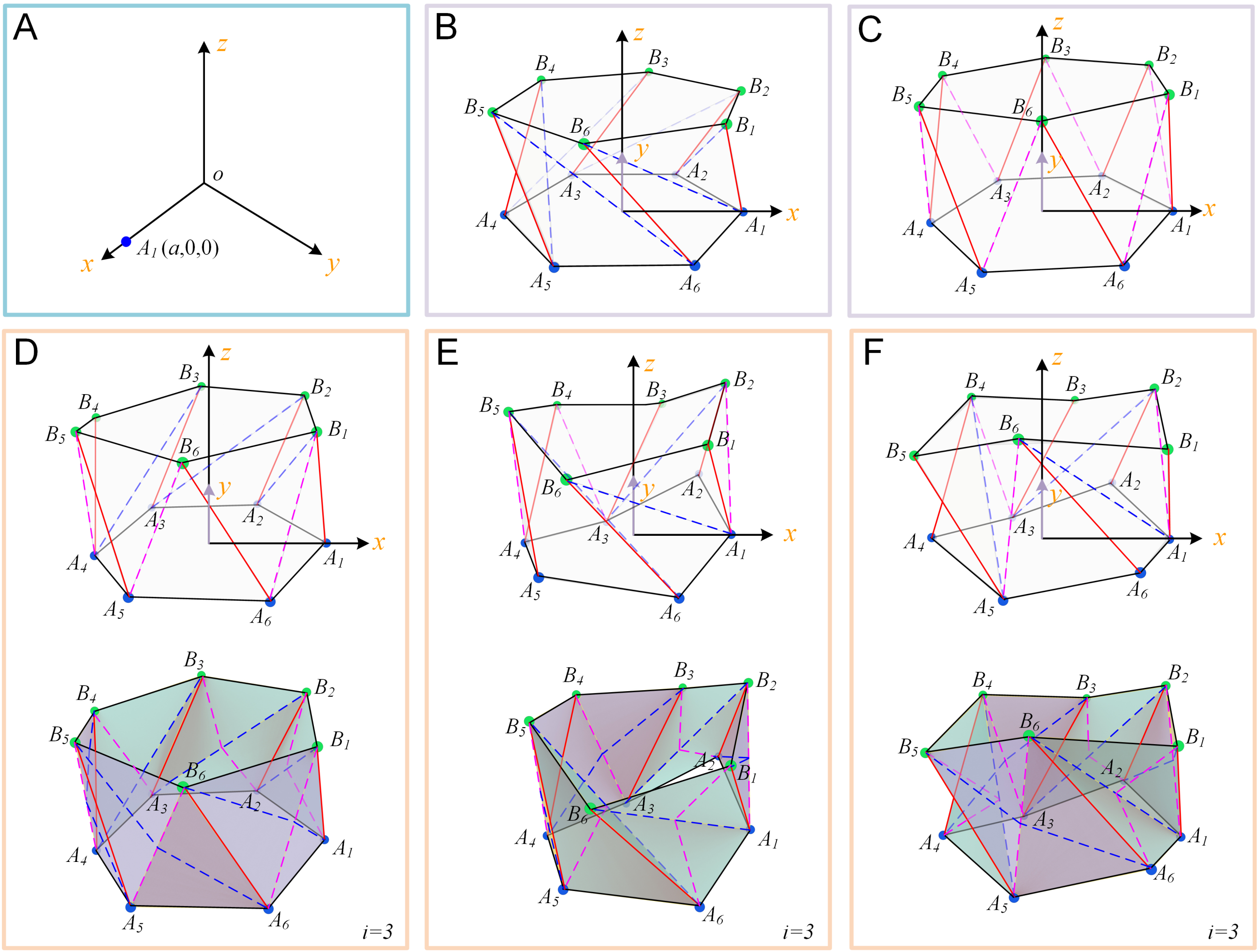

Fig. S9. The results of the stable states using the point-searching method. (A) The initial point and coordinate system of the point-searching method. (B) The structural form of the second stable state obtained from the point-searching method. (C) The structural form of the third stable state obtained from the point-searching method. (D-F) The structural forms of the special stable states (i=3) obtained from the point-searching method, including three unit cells with the second stable state and others with the third stable state.

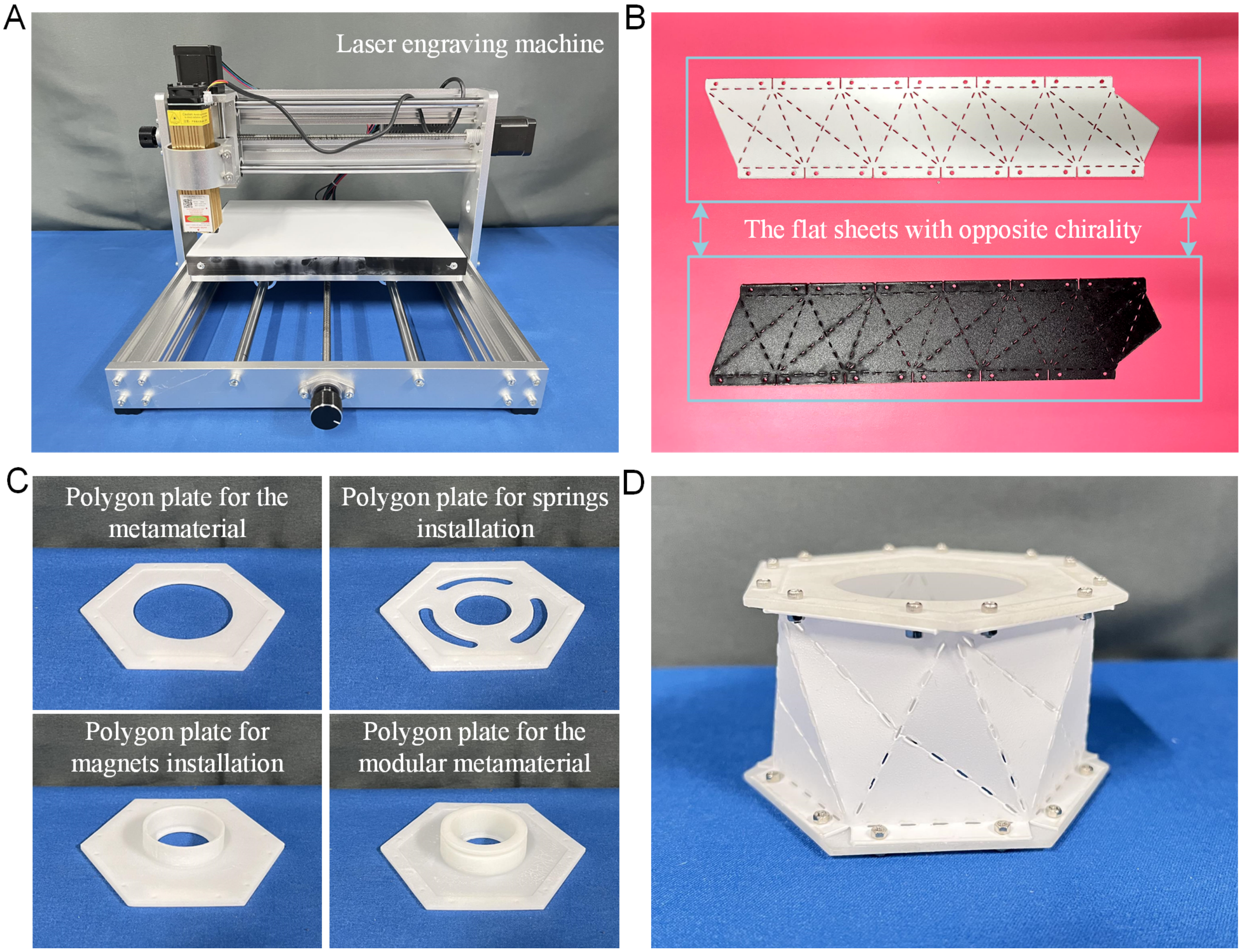

Fig. S10. The fabrication method of the metamaterial. (A) Laser engraving machine and the polypropylene material. (B) The polypropylene material with the laser engraved pattern. (C) Top/bottom polygon panels (3D printed resin material). (D) The metamaterial sample. M1.4*5 mm bolts were used to connect the polypropylene sheet to the polygon plates.

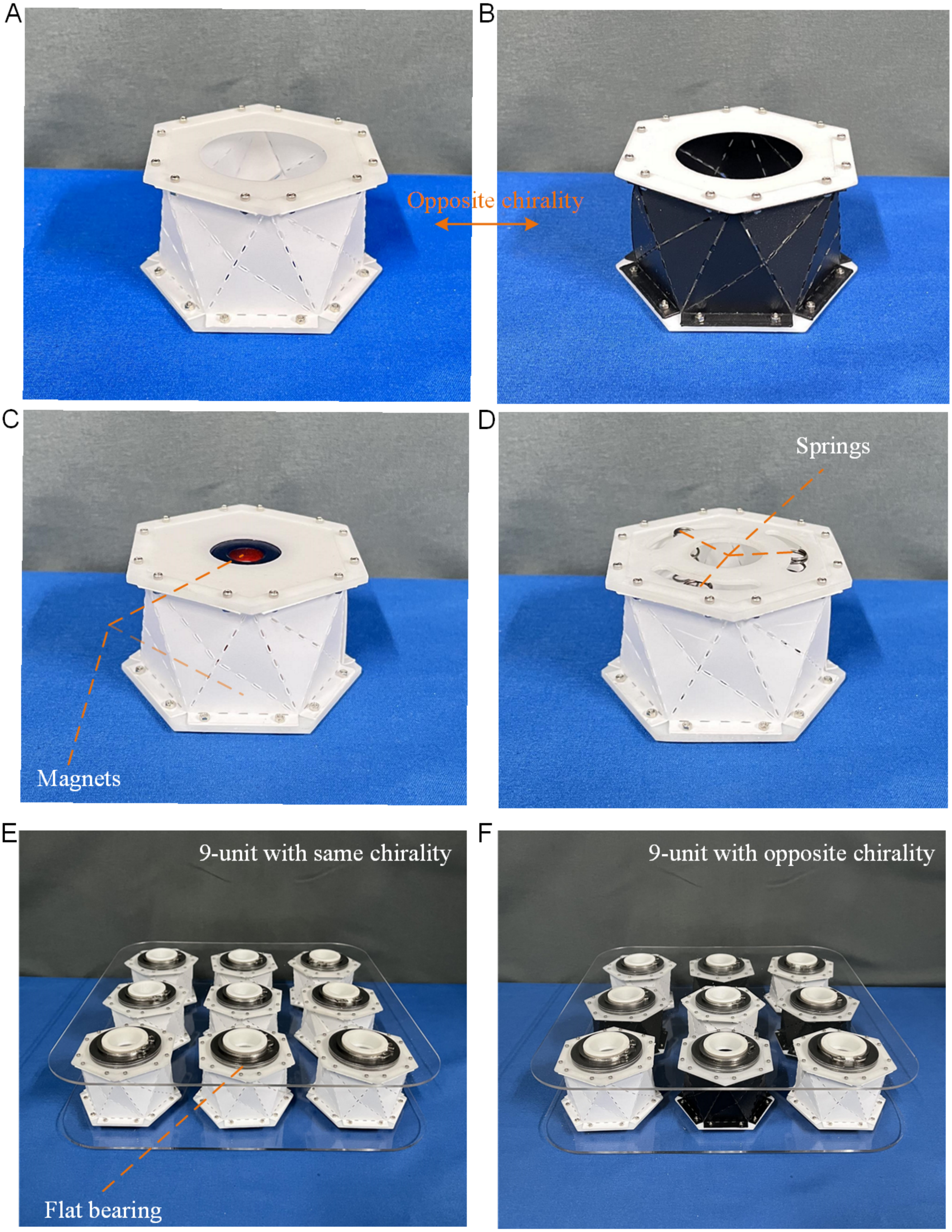

Fig. S11. Photos of the metamaterials. (A) The single-module metamaterial. (B) The single-module metamaterial with opposite chirality. (C) The single-module metamaterial with reverse-arranged magnets. (D) The single-module metamaterial with built-in springs. (E) Modular metamaterial with same chirality. (F) Modular metamaterial with opposite chirality. The flat bearings were placed on the top/bottom of the modular metamaterials to ensure the rotational DOF.

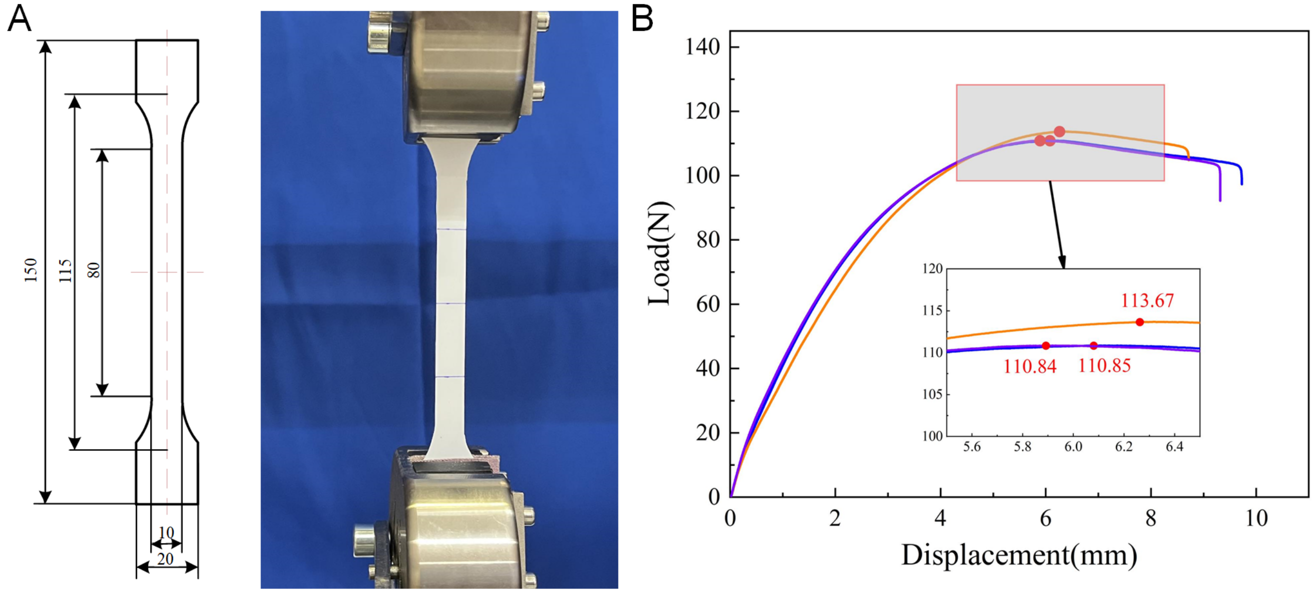

Fig. S12. Performance measurement and data analysis of the polypropylene material. (A) The polypropylene material sample and the initial state of the tensile experiments. (B) The load/displacement curves of the tensile experiments.

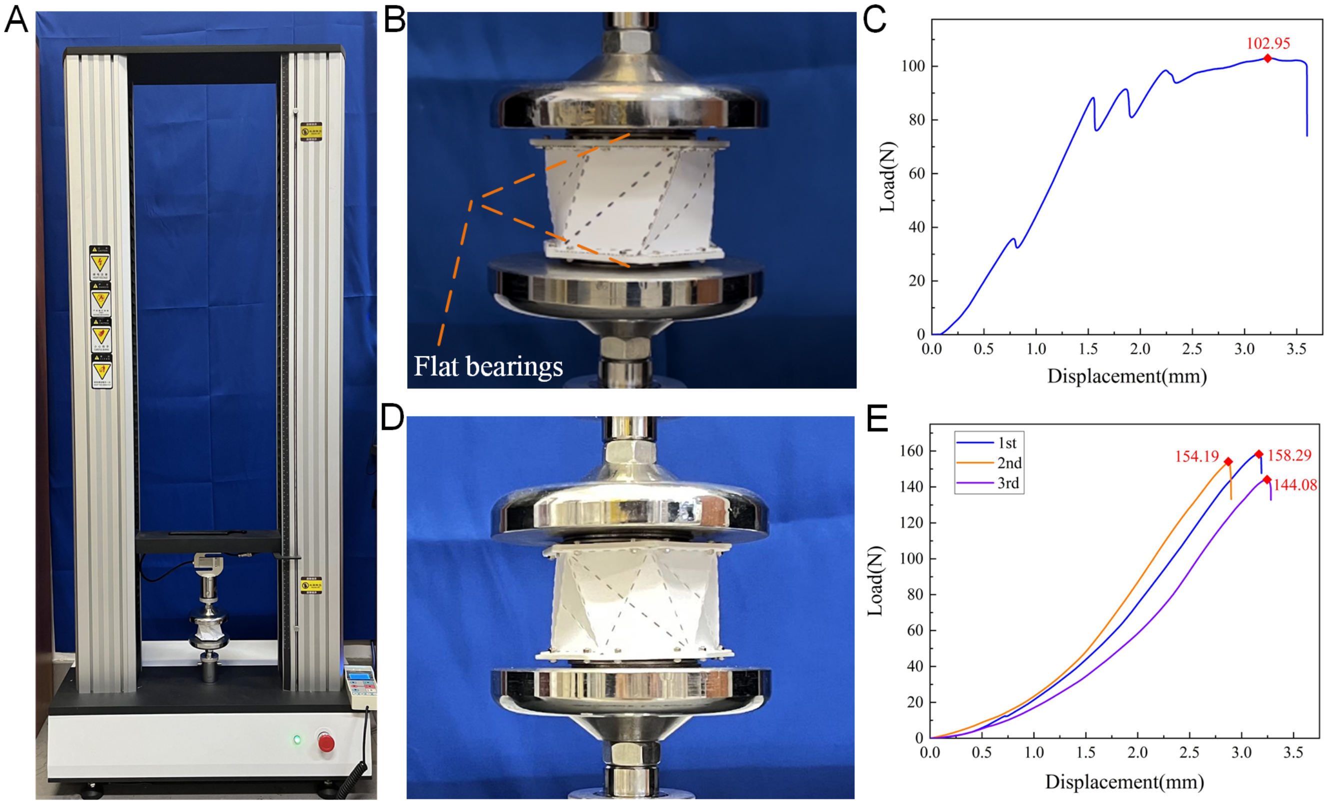

Fig. S13. Explanation for the tunable stiffness of the metamaterials. (A) Universal tensile testing machine and the metamaterial sample. (B) Compression experimental photo of the TCO-inspired metamaterial at the third stable state. (C) Load/displacement curve of the TCO-inspired metamaterial at the third stable state. (D) Compression experimental photo of the MTCO-inspired metamaterial at the third stable state. (E) Three consecutive load/displacement curves of the MTCO-inspired metamaterial at the third stable state. Flat bearings are placed between the sample and the fixtures to ensure the rotation DOF of the metamaterials. And the experimental data show that the TCO-inspired metamaterial has poorer stability than the MTCO-inspired metamaterial.

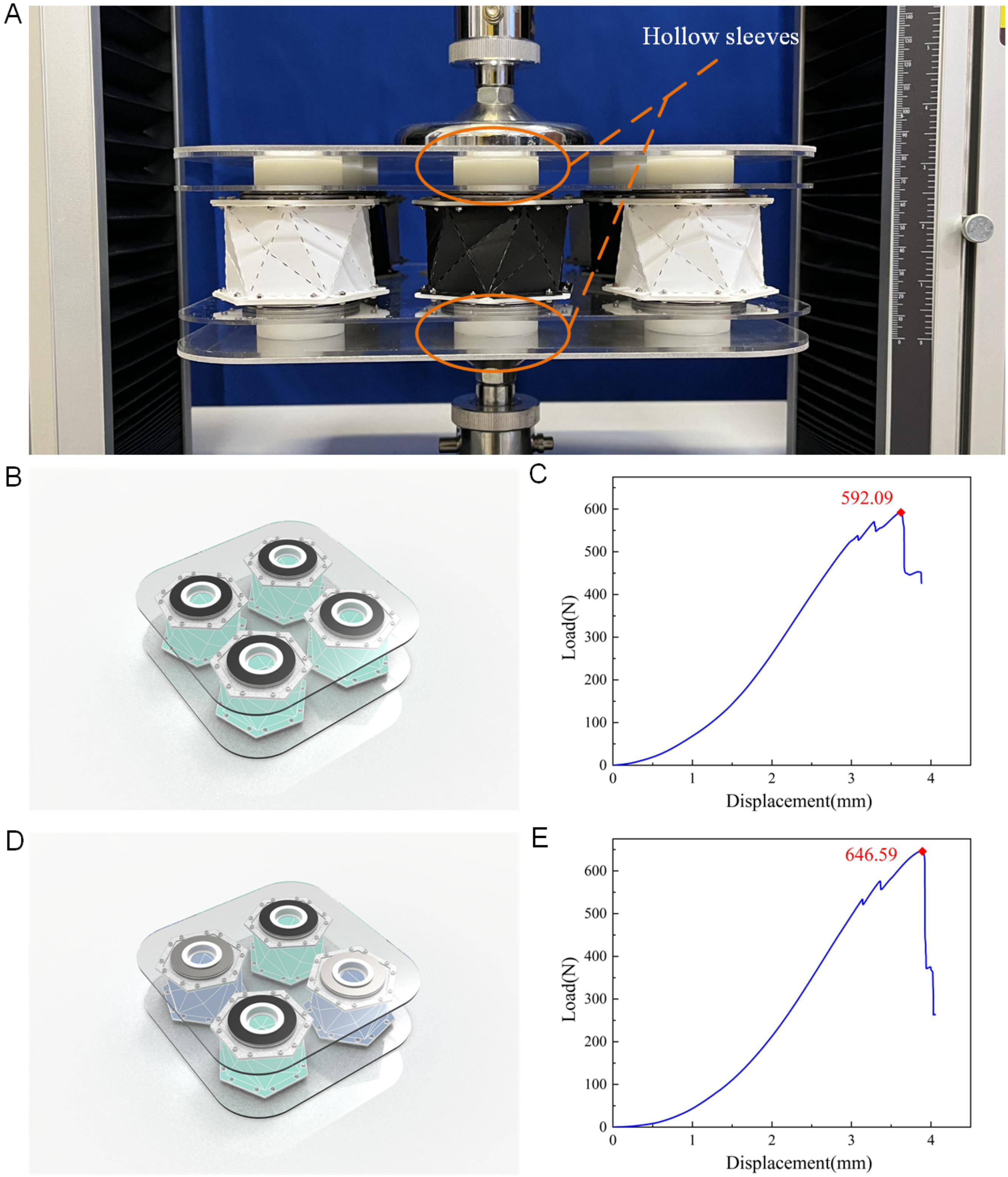

Fig. S14. Photos of the compression experiments and data analysis. (A) Compression experiment photo of the modular metamaterials with opposite chirality (9-unit). (B) The modular metamaterials with same chirality (4-unit). (C) Load/displacement curve for the modular metamaterial with same chirality. (D) The modular metamaterials with opposite chirality (4-unit). (E) Load/displacement curve for the modular metamaterial with opposite chirality. During the compression experiments, the hollow sleeves were arranged between the modular metamaterials and the fixtures, concentrating the pressure on the metamaterials.

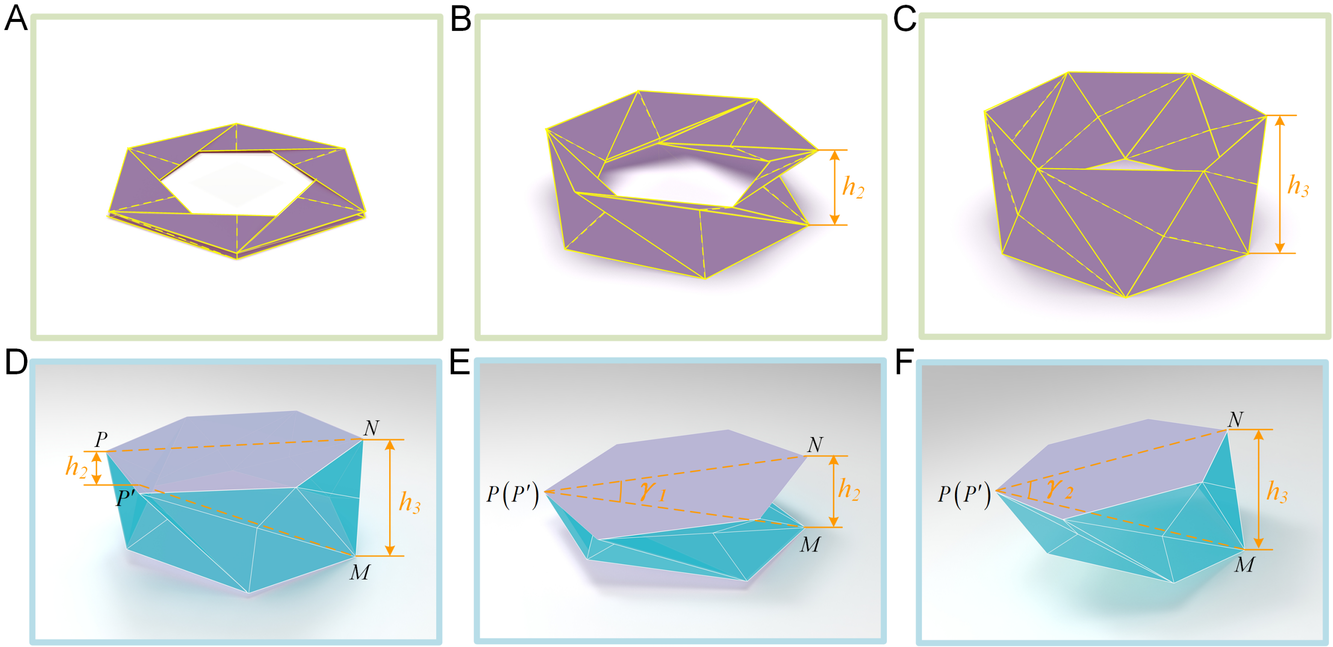

Fig. S15. Analysis on the motion characteristics of the multi-triangles cylindrical origami. (A-C) Analysis of the stretchable range of the MTCO. (A) Initial position of the zero-stiffness property. (B) Termination position of the zero-stiffness property (second stable state). (C) The third stable state. With the proposed third stable state, the stretchable range of the MTCO is extended. **(D-F) Analysis of the bendable angles of the MTCO.** (D) One unit cell is at the third stable state, and the diagonal cell is at the termination position of the zero-stiffness property. (E) One unit cell is at the termination position of the zero-stiffness property, and the diagonal cell is at the initial position of the zero-stiffness property, the bending angle could reach $\gamma_1 \approx 14.65°$. (F) One unit cell is at the third stable state, and the diagonal cell is at the initial position of the zero-stiffness property, the bending angle could reach $\gamma_2 \approx 25.54°$. Similarly, the bending degree of the MTCO is expanded.

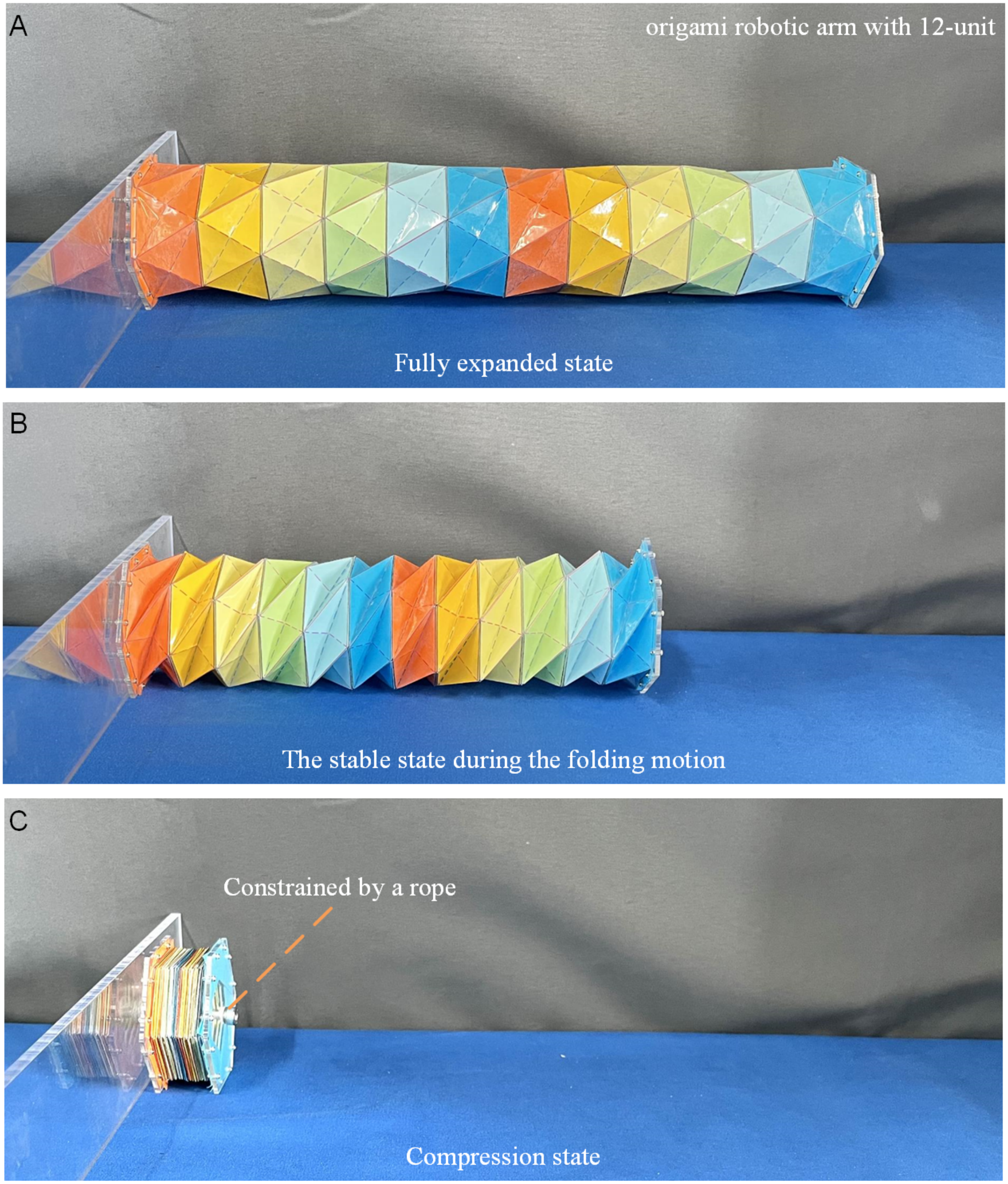

Fig. S16. Three folding states of the origami-inspired robotic arm (12 origami units). (A) Fully expanded steaty of the origami robotic arm. (B) The stable state during the folding motion of the origami robotic arm. (C) Compression state of the origami robotic arm, constrained by a rope. With the third stable state of the MTCO, the stretchable range of the origami robotic arm is extended.

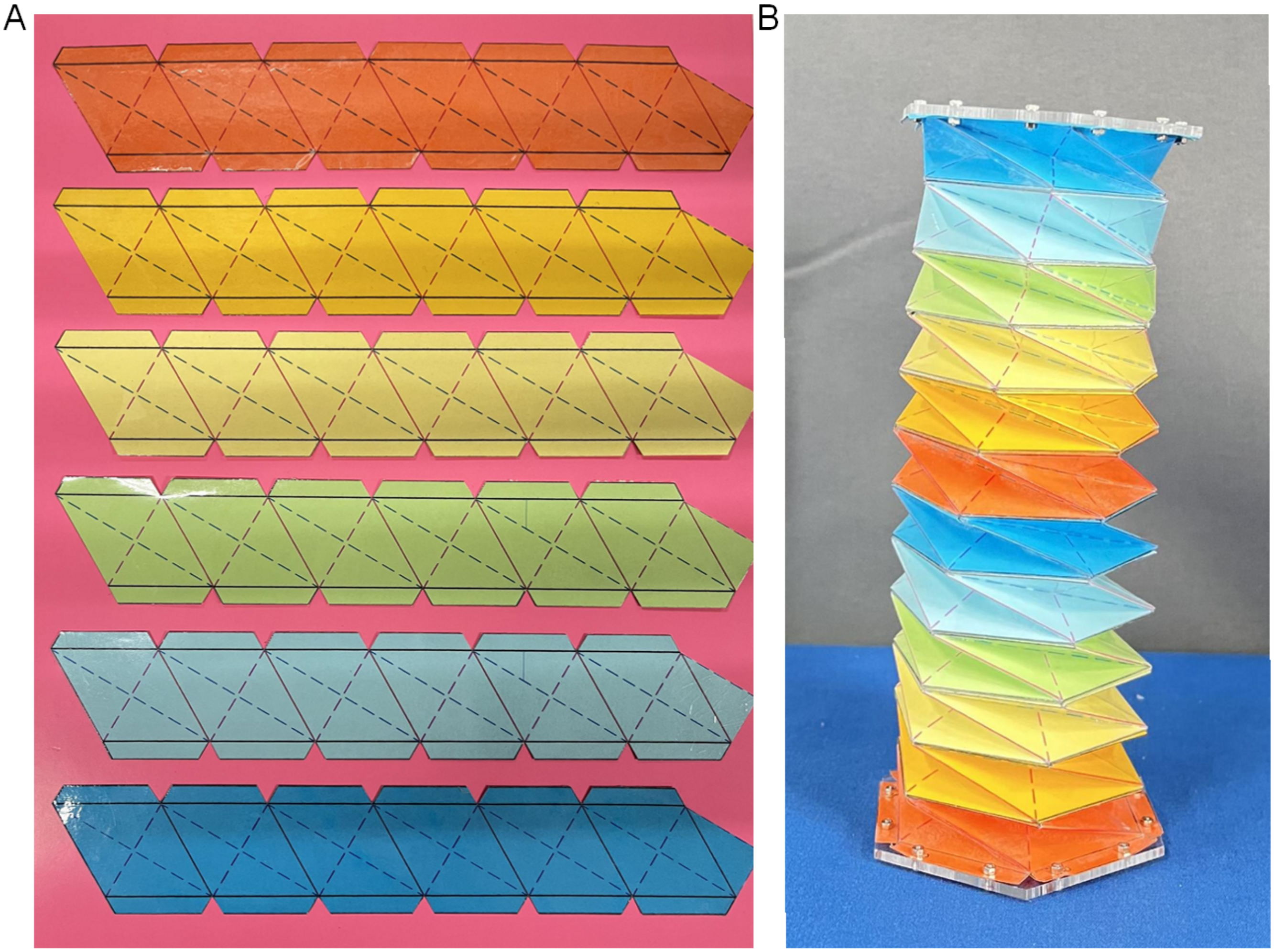

Fig. S17. Fabrication of the origami-inspired robotic arm sample. (A) The MTCO pattern covered with the film on the outer surface. (B) Photo of the origami-inspired robotic arm sample, consisting of 12 origami units. The top/bottom polygonal material was cut down and glued together with double-sided tape, and two Plexiglas panels were connected to the ends of the robotic arm.

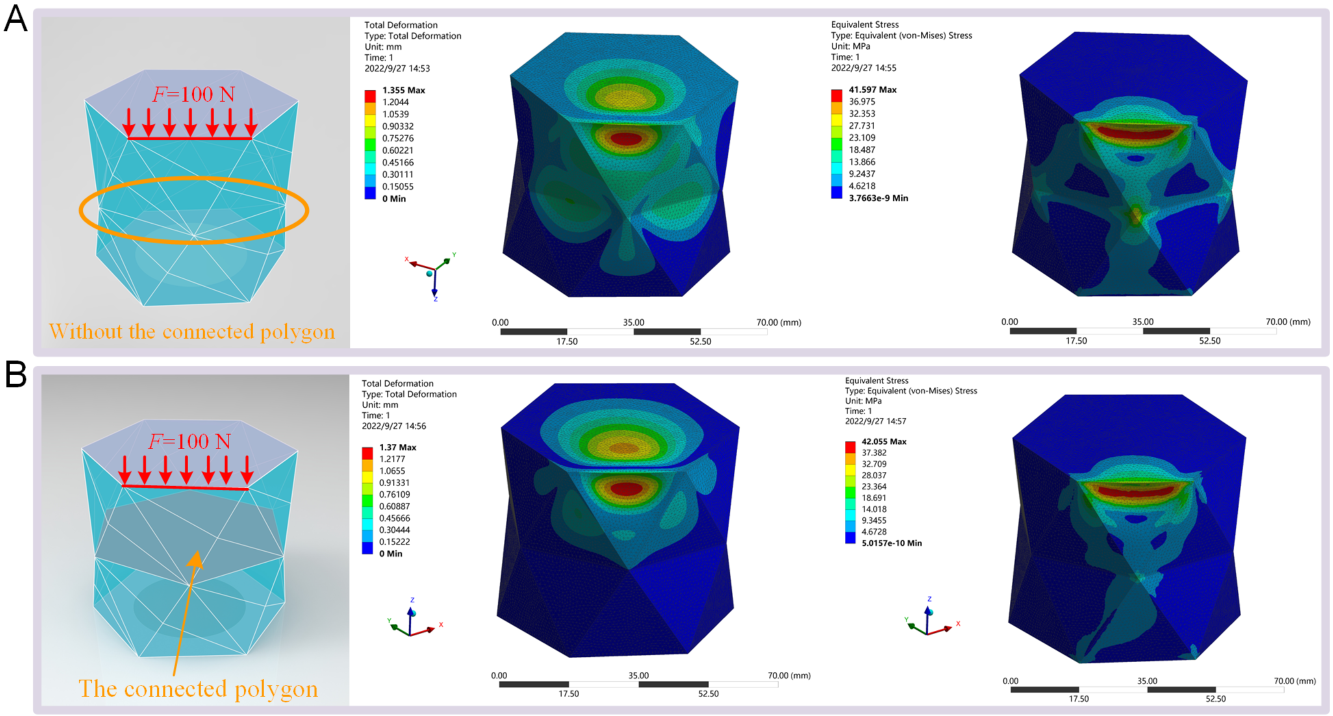

Fig. S18. Deformation and stress analysis of the multi-unit origami with/without the connected polygon. (A) Deformation and stress analysis of the multi-unit origami without the connected polygon. (B) Deformation and stress analysis of the multi-unit origami with the connected polygon. This results show that the multi-unit robotic arm is more likely to undergo the desired motion with the same driving force when the internal connecting material is removed.

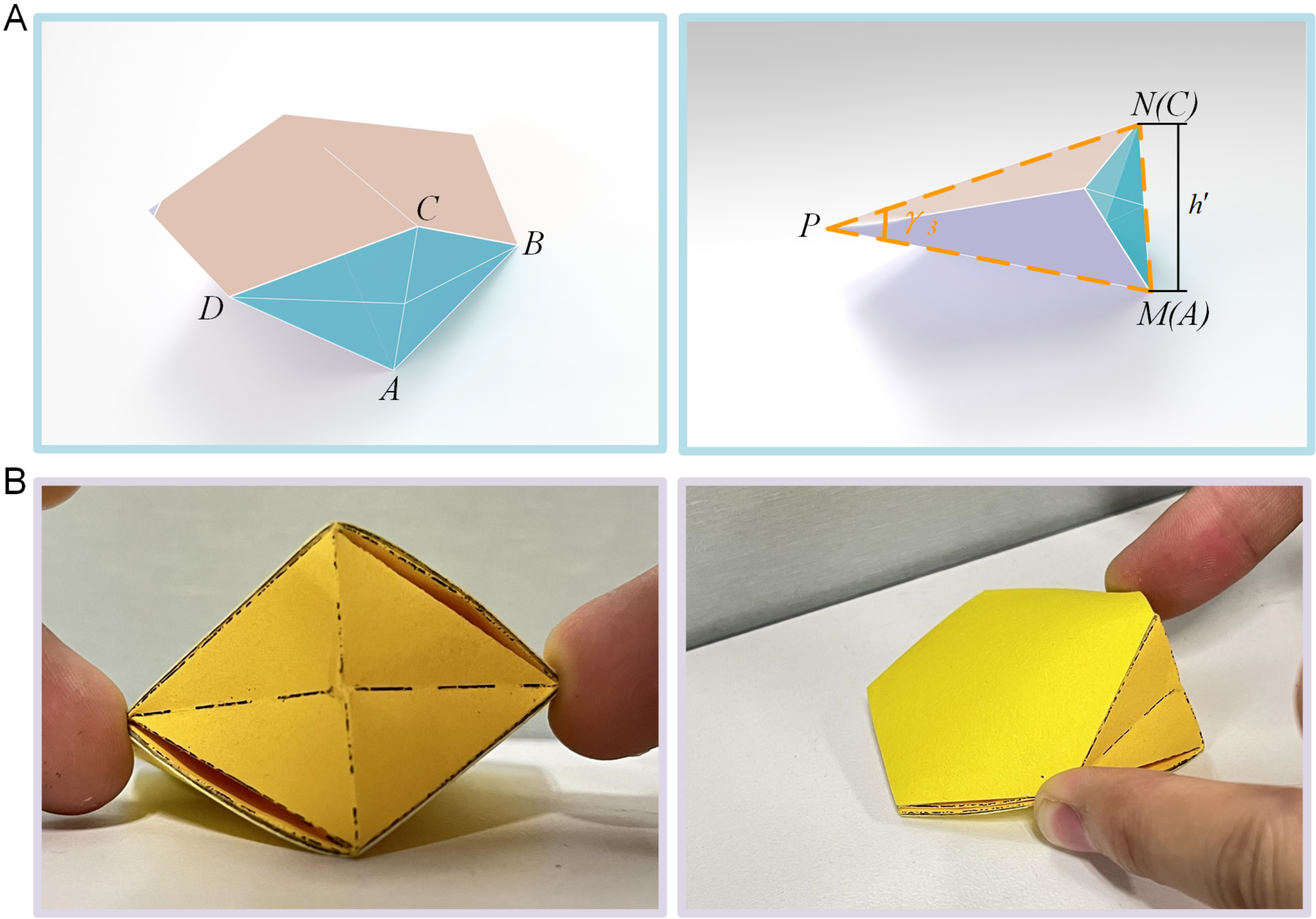

Fig. S19. Analysis of the maximum bending angle by applying an external force, $\gamma_3 \approx 37.17^\circ$. (A) Side view and front view of the maximum bending angle model. (B) Photos of the maximum bending angle sample.

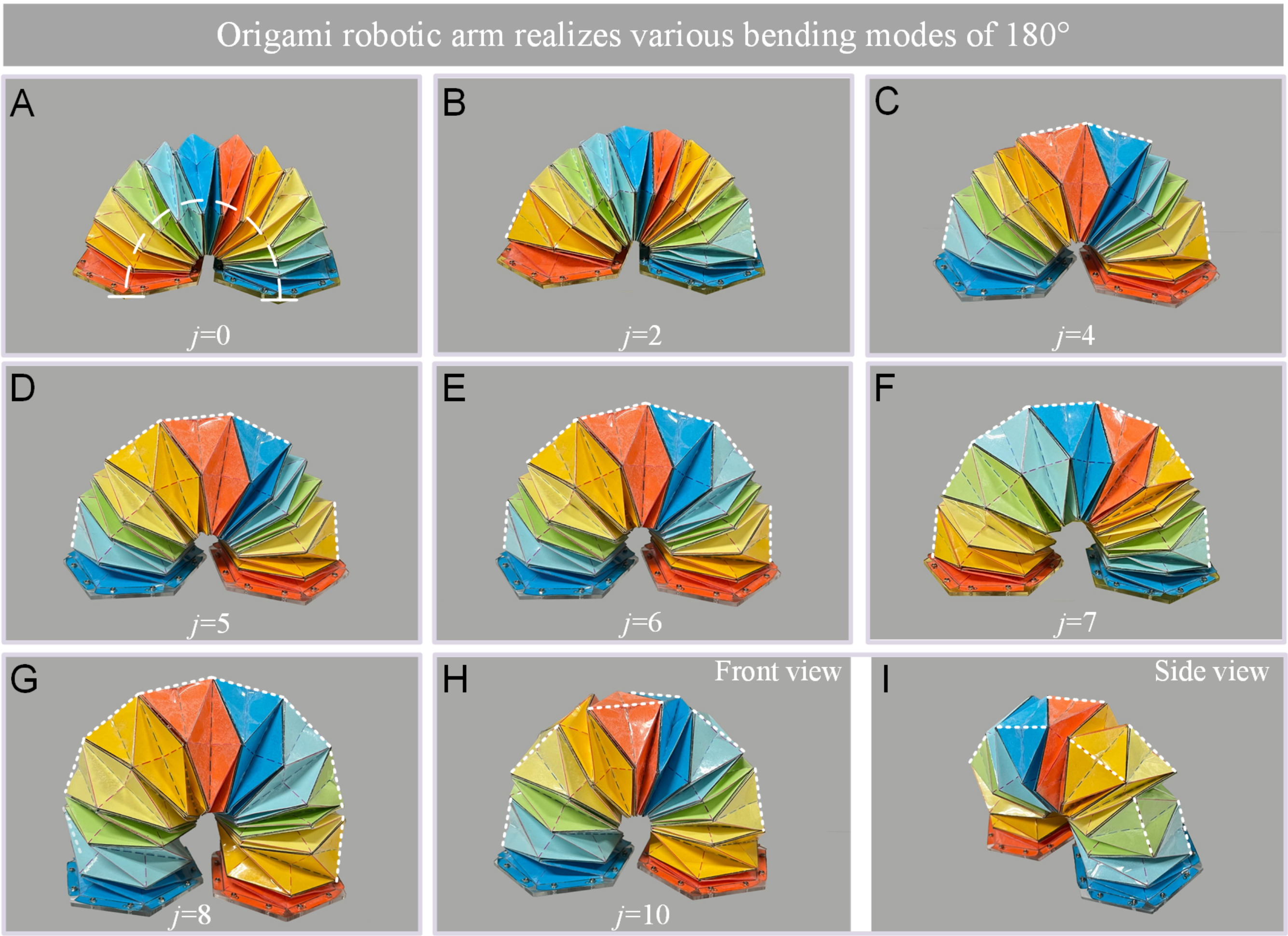

Fig. S20. Multiple bending modes of the origami-inspired robotic arm (12-unit). Without the proposed tristable property, the origami robotic arm achieves a bending angle of 180° by only one mode ($j = 0$), as shown in Fig. A. However, with the proposed tristable property of the multi-triangles cylindrical origami, the motion modes of the origami robotic arm are more abundant. j indicates the number of the unit cells in the form of the third stable state.

Table S1. The instructions for the folding process of the multi-triangles cylindrical origami

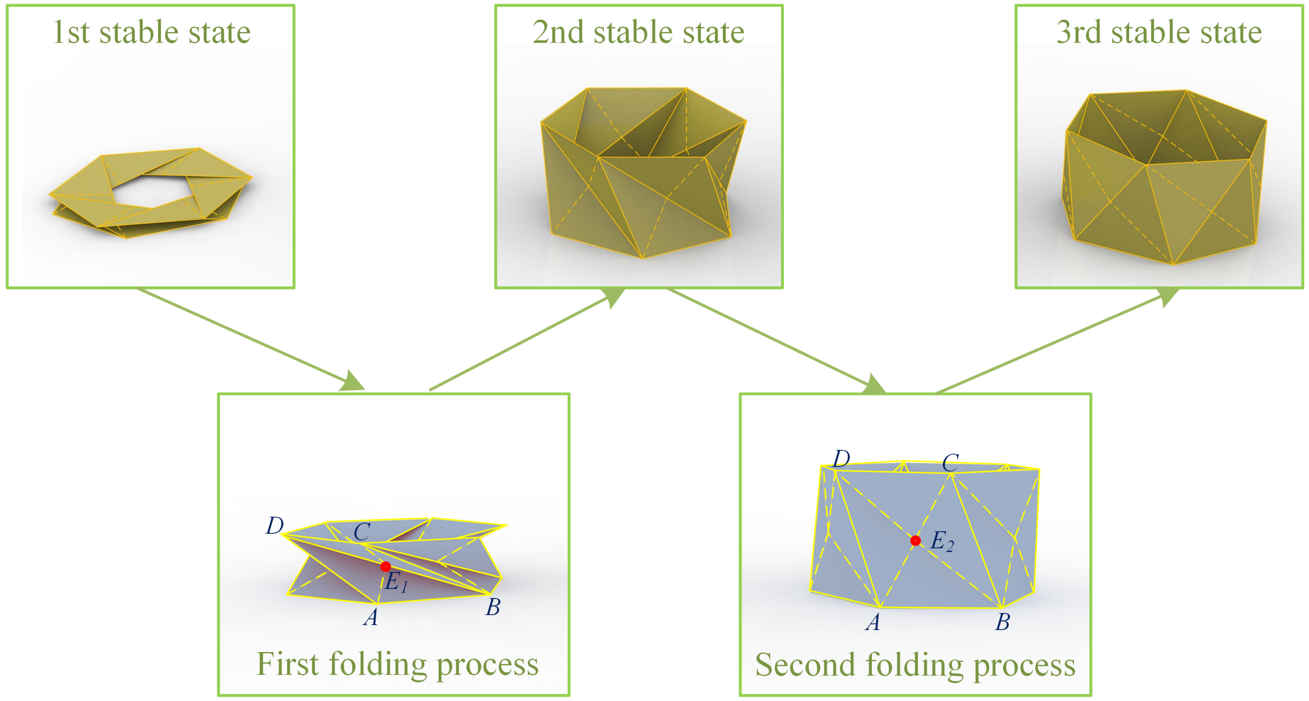

Active crease line:<br>The valley crease lines BD	Active crease line:<br>No
$BD = BE_1 + DE_1$<br>$AC = AE_1 + CE_1$<br>Point E_1 is the midpoint of crease line BD	$BD = BE_2 + DE_2$<br>$AC = AE_2 + CE_2$<br>Point E_2 is a moving point
Truss model: $E = 1/2k(\Delta L_{AD}^2 + \Delta L_{BD}^2 + \Delta L_{AC}^2)$	
$E = 1/2k(\Delta L_{AD}^2 + \Delta L_{BD}^2 + \Delta L_{AE_1}^2 + \Delta L_{CE_1}^2)$	$E = 1/2k(\Delta L_{AD}^2 + \Delta L_{BE_2}^2 + \Delta L_{DE_2}^2 + \Delta L_{AE_2}^2 + \Delta L_{CE_2}^2)$
Characteristics: The unit cell is divided into two triangular panels ΔABD and ΔBCD.	Characteristics: The unit cell is divided into four triangular panels ΔABE_2, ΔBCE_2, ΔCDE_2, and ΔADE_2.

Table S2. The algorithm for the modified truss model

Algorithm for the modified truss model:	
1	Input α, β, and a;
2	Calculate the lengths of the crease lines AD, AC, and BD at the plane state;
3	Set up the coordinate system and give the coordinates of points A, B, C, and D;
4	Pre-judgment the property of the multi-triangles cylindrical origami with current geometric parameters using the equation $E = 1/2k(\Delta L_{AD}^2 + \Delta L_{BD}^2)$;
5	Finding the demarcation point of the two folding processes: if it is monostable determined by step 4, the demarcation point is the steady-state position; if it is bistable, the demarcation point is the second steady-state position; if it is a zero-stiffness property, the demarcation point is the termination position of the zero-stiffness property;
6	The folding motion of the multi-triangles cylindrical origami is divided into two processes using the demarcation points obtained in step 5;
7	The first folding process is calculated using $E = 1/2k(\Delta L_{AD}^2 + \Delta L_{BD}^2 + \Delta L_{AE_1}^2 + \Delta L_{CE_1}^2)$. Point E_1 is the midpoint of the crease line BD, then the length of BD can be expressed by the coordinates of points B and D;
8	The second folding process is calculated using $E = 1/2k(\Delta L_{AD}^2 + \Delta L_{BE_2}^2 + \Delta L_{DE_2}^2 + \Delta L_{AE_2}^2 + \Delta L_{CE_2}^2)$. Point E_2 is a moving point;
9	Synthesize the energy change curves of the two folding processes to obtain the final energy landscape.

Table S3. The coordinates of the vertexs corresponding to the second stable state of the MTCO

	The point-searching method (mm)			The modified truss model (mm)		
	X	Y	Z	X	Y	Z
A1	30	0	0	30	0	0
A2	15.2615	26.1168	-0.8272	15	25.9808	0
A3	-14.7381	26.0426	-0.9523	-15	25.9808	0
A4	-29.7751	0.0832	-0.8639	-30	3.67E-15	0
A5	-14.8822	-25.9568	-0.5206	-15	-25.9808	0
A6	15.1134	-26.0457	-0.0092	15	-25.9808	0
B1	20.9171	-20.0840	29.6421	21.5436	20.8776	29.3040
B2	29.0186	8.7871	28.7524	28.8523	-8.2185	29.3040
B3	7.2305	29.4089	28.5539	7.3087	-29.0961	29.3040
B4	-21.5923	21.0906	28.3980	-21.5436	-20.8776	29.3040
B5	-29.1608	-7.9391	28.3942	-28.8523	8.2185	29.3040
B6	-7.7372	-28.9345	28.8708	-7.3087	29.0961	29.3040

Table S4. The coordinates of the vertexs corresponding to the third stable state of the MTCO

	The point-searching method (mm)			The modified truss model (mm)		
	X	Y	Z	X	Y	Z
A1	30	0	0	30	0	0
A2	15.0119	26.3095	-1.3806	15	25.9808	0
A3	-15.2518	26.4528	-3.0616	-15	25.9808	0
A4	-30.5271	0.2742	-3.3641	-30	3.67E-15	0
A5	-15.5386	-26.0353	-1.9874	-15	-25.9808	0
A6	14.7249	-26.1786	-0.3040	15	-25.9808	0
B1	23.6758	-14.9436	33.9178	26.4686	14.1214	34.0560
B2	24.4381	15.3518	33.3296	25.4638	-15.8617	34.0560
B3	-1.3753	31.1413	31.5678	-1.0048	-29.9832	34.0560
B4	-27.9581	16.6250	30.3965	-26.4686	-14.1214	34.0560
B5	-28.7202	-13.6704	30.9835	-25.4638	15.8618	34.0560
B6	-2.9067	-29.4601	32.7428	1.0048	29.9832	34.0560

Table S5. The coordinates of the vertexs corresponding to the special stable states (i=3) of the MTCO

	The point-searching method (mm)								
	X	Y	Z	X	Y	Z	X	Y	Z
A1	30	0	0	30	0	0	30	0	0
A2	14.8462	26.3433	-0.4625	24.6074	29.1060	5.7649	18.5875	27.6366	4.4631
A3	-15.5565	26.1777	-1.5251	-2.0471	24.6063	-7.4419	-9.8475	23.2195	-4.4129
A4	-28.1903	-1.1610	-4.1028	-21.9694	2.1774	-3.3607	-28.7738	0.1391	0.3857
A5	-16.0847	-28.2306	1.3563	-14.4296	-26.6000	2.3091	-10.0270	-23.1156	-3.4372
A6	13.7272	-25.4705	-1.3534	15.2608	-26.1337	-2.9453	18.5141	-27.2627	5.6267
B1	22.8826	-16.0227	33.3509	20.5441	-20.6990	29.4036	23.3967	-21.0093	30.0653
B2	24.7543	14.3101	33.8405	27.6186	7.8980	36.1860	25.3137	8.6122	35.7959
B3	-1.1562	29.8991	32.8355	13.8501	32.4890	25.4423	7.6841	31.5384	27.4042
B4	-28.6352	19.2375	26.9241	-14.6438	22.4283	27.4014	-19.5921	19.0536	31.1562
B5	-27.9999	-10.5197	31.7424	-22.1528	-6.3884	32.7512	-29.7996	-8.5013	24.5696
B6	-4.3723	-29.3637	31.1906	-7.2814	-31.6582	26.0197	-6.2297	-25.2359	33.4215

Movie S1. Structural characteristics and folding motion of the multi-triangles cylindrical origami.

Movie S2. The three stable states of Kresling pattern origami and their folding motions.

Movie S3. High stiffness test processes for the third stable state.

Movie S4. The special stable states of the multi-triangles cylindrical origami.

Movie S5. The another special stable state of the multi-triangles cylindrical origami.

Movie S6. The multiple properties of the origami-inspired metamaterials.

Movie S7. Origami-inspired robotic arm and the features demonstration.

References

1. Masana, R., Daqaq, M. F., Equilibria and bifurcations of a foldable paper-based spring inspired by Kresling-pattern origa. *Phys. Rev. E* **100**, 063001 (2019).
2. Lu, L., Dang, X. X., Feng, F., et al., Conical Kresling origami and its applications to curvature and energy programming. *P. Roy. Soc. A* **477**, 20210712 (2022).
3. Zhai, Z. R., Wang, Y., Jiang, H. Q., Origami-inspired, on-demand deployable and collapsible mechanical metamaterials with tunable stiffness. *PNAS* **115**, 2032-2037 (2018).
4. Yasuda, H., Tachi, T., Lee, M., Yang, J., Origami-based tunable truss structures for non-volatile mechanical memory operation. *Nat. Commun.* **8**, 962 (2017).
5. Ma, J. Y., Song, J. C., Chen, Y., An origami-inspired structure with graded stiffness. *Int. J. Mech. Sci.* **136**, 134-142 (2018).
6. Nauroze, S. A., Novelino, L. S., Tentzeris, M. M., et al., Continuous-range tunable multilayer frequency-selective surfaces using origami and inkjet printing, *PNAS* **115**: 13210–13215 (2018).
7. Fang, H., Li, S., Ji, H., Wang, K., Dynamics of a bistable Miura-origami structure. *Phys. Rev. E* **95**, 052211 (2017).
8. Cai, J., Deng, X., Feng, J., et al., Geometric Design and Mechanical Behavior of a Deployable Cylinder with Miura Origami. *Smart Mater. Struct.* **24**, 125031 (2015).
9. Suh, J. , Kim, T. H. , Han, J. H., New Approach to Folding a Thin-Walled Yoshimura Patterned Cylinder. *J. Spacecraft Rockets* **58**, 516-530 (2020).
10. Kidambi N., Wang K. W., Dynamics of Kresling origami deployment. *Phys. Rev. E* **101**, 063003 (2020).
11. Zhai, Z. R., Wu, L. L., Jiang, H. Q., Mechanical metamaterials based on origami and kirigami. *Appl. Phys. Rev.* **8**, 041319 (2021).
12. Wu, S., Ze, Q. J., Dai, J. Z., et al., Stretchable origami robotic arm with omnidirectional bending and twisting. *PNAS* **118**, e2110023118 (2021).
